# Effects of cognitive behaviour therapy for worry on persecutory delusions in patients with psychosis (WIT): a parallel, single-blind, randomised controlled trial with a mediation analysis

**DOI:** 10.1016/S2215-0366(15)00039-5

**Published:** 2015-03-31

**Authors:** Daniel Freeman, Graham Dunn, Helen Startup, Katherine Pugh, Jacinta Cordwell, Helen Mander, Emma Černis, Gail Wingham, Katherine Shirvell, David Kingdon

**Affiliations:** a Department of Psychiatry, University of Oxford, Oxford, UK; b Centre for Biostatistics, Institute of Population Health, University of Manchester, Manchester, UK; c Medical Research Council NorthWest Hub for Trials Methodology Research, Department of Biostatistics, University of Liverpool, Liverpool, UK; d Sussex Partnership NHS Foundation Trust, Research and Development Department, Mill View Hospital, Hove, UK; e Academic Department of Psychiatry, Faculty of Medicine, University of Southampton, Southampton, UK

## Abstract

**Background:**

Worry might be a contributory causal factor in the occurrence of persecutory delusions in patients with psychotic disorders. Therefore we postulated that reducing worry with cognitive behaviour therapy (CBT) would reduce persecutory delusions.

**Methods:**

For our two-arm, assessor-blinded, randomised controlled trial (Worry Intervention Trial [WIT]), we recruited patients aged 18–65 years with persistent persecutory delusions but non-affective psychosis from two centres: the Oxford Health National Health Service (NHS) Foundation Trust (Oxford, UK) and the Southern Health NHS Foundation Trust (Southampton, UK). The key inclusion criteria for participants were a score of at least 3 on the Psychotic Symptoms Rating Scale (PSYRATS) denoting a current persecutory delusion; that the delusion had persisted for at least 3 months; a clinical diagnosis of schizophrenia, schizoaffective disorder, or delusional disorder; and a clinically significant level of worry. We randomly assigned (1:1) eligible patients, using a randomly permuted block procedure with variable block sizes and division by four strata, to either six sessions of worry-reduction CBT intervention done over 8 weeks added to standard care (the CBT-intervention group), or to standard care alone (the control group). The assessors were masked to patient allocations and did their assessments at week 0 (baseline), 8 weeks (end of treatment), and 24 weeks, follow-up. The primary outcomes were worry measured by the Penn State Worry Questionnaire (PSWQ) and delusions measured by the PSYRATS-delusion scale; we did the analyses in the intention-to-treat population, and also did a planned mediation analysis. This trial is registered with the ISRCTN Registry (number ISRCTN23197625) and is closed to new participants.

**Findings:**

From Nov 1, 2011, to Sept 9, 2013, we recruited 150 eligible participants and randomly assigned 73 to the CBT intervention group, and 77 to the control group. 143 patients (95%) provided primary outcome follow-up data. Compared with standard care alone, at 8 weeks the CBT intervention significantly reduced worry (mean difference 6·35 [SE 1·56] PSWQ units, 95% CI 3·30–9·40; p<0·001) and persecutory delusions (2·08 [SE 0·73] PSYRATS units, 95% CI 0·64–3·51; p=0·005). The reductions were maintained to 24 weeks follow-up. The mediation analysis suggested that the change in worry accounted for 66% of the change in delusion. No patients died or were admitted to secure units during our study. Six suicide attempts (two in the CBT intervention group, and four in the control group) and two serious violent incidents (one in each group) were noted, but no adverse events were deemed related to the treatments or the assessments.

**Interpretation:**

To our knowledge, this is the first large trial focused on persecutory delusions. We have shown that long-standing delusions were significantly reduced by a brief intervention targeted on worry, although the limitations for our study include no determination of the key elements within the intervention. Our results suggest that worry might cause paranoia, and that worry intervention techniques might be a beneficial addition to the standard treatment of psychosis.

**Funding:**

Efficacy and Mechanism Evaluation programme, which is a UK Medical Research Council and National Institute of Health Research partnership.

## Introduction

Treatments for psychotic disorders such as schizophrenia need substantial improvement. Our approach is to study single psychotic experiences such as persecutory delusions, establish a theoretical model, and translate the knowledge gained into treatment. To build the treatment, one putative causal factor is taken at a time, changed, and the effect on the psychotic occurrence examined.^1^ This approach is called an interventionist-causal model approach.^2^ In this Article, we report the effects of targeting one causal factor—worry—in patients with persecutory delusions.

Worry is an expectation of the worst happening. It consists of repeated negative thoughts about potential adverse outcomes, and is a psychological component of anxiety. Worry brings implausible ideas to mind, keeps them there, and increases the level of distress. Therefore we have postulated that worry is a causal factor in the development and maintenance of persecutory delusions, and have tested this theory in several studies.^3^, ^4^, ^5^, ^6^, ^7^, ^8^, ^9^ We showed that levels of worry in patients with persecutory delusions are similar to those noted in generalised anxiety disorder;^3^ a dose-response association exists between levels of worry and paranoia;^4^ worry is a predictor of the occurrence and persistence of non-clinical paranoia in the general population^5^, ^6^ and in experimental settings;^7^ and levels of worry predict the persistence of persecutory delusions.^8^, ^9^ Other study groups are also replicating and extending these findings.^10^, ^11^ We have translated this knowledge into treatment and shown in a pilot trial^12^ that a brief intervention of worry-reduction added to standard care might lead to reductions in both worry and persecutory delusions. In the terminology of the scientific literature, worry in delusions is a so-called inus condition—“an insufficient but non-redundant part of an unnecessary but sufficient disorder.”^13^ Persecutory delusions arise from a combination of causes, with each causal factor increasing the probability of such fears occurring.

We planned our trial as a rigorous test of these mechanistic links to inform both theory and treatment. A key mechanism (worry) was targeted. The appropriate control condition was a standard care group to establish that the mechanism had been successfully targeted, which would then allow examination of the effects of the mechanism change on the central clinical occurrence (persecutory delusions). We planned an elaborate mediation analysis to substantiate the postulated mechanism of delusion change. The aim of our study was to investigate whether the intervention with cognitive behaviour therapy (CBT) would reduce levels of worry in patients with persecutory delusions and reduce the delusions themselves; the improvements would be maintained at follow-up; and the reduction in worry would mediate changes in persecutory delusions.

## Methods

### Study design and participants

We did a randomised, controlled, single-blind trial in two UK centres: the Oxford Health National Health Service (NHS) Foundation Trust, Oxford, and the Southern Health NHS Foundation Trust, Southampton. These large mental health services cover populations of about 1·2 million people each. The trial received a favourable opinion from an NHS Research Ethics Service Committee, and the trial protocol has been published.^14^ We sought referrals of patients aged 18–65 years with persecutory delusions from both centres. The inclusion criteria were: a current persecutory delusion as defined by Freeman and Garety,^15^ scoring at least 3 on the conviction scale of the Psychotic Symptoms Rating Scale (PSYRATS);^16^ that the delusion had persisted for at least 3 months; a clinical diagnosis of schizophrenia, schizoaffective disorder, or delusional disorder (ie, a diagnosis of non-affective psychosis); and a clinically significant level of worry, as shown by a score of more than 44 on the Penn State Worry Questionnaire (PSWQ).^17^ Where major changes in drugs were going to be made, entry to the investigation would not occur until at least 1 month after stabilisation of dosage. Criteria for exclusion were: a primary diagnosis of alcohol or substance dependency or personality disorder; an organic syndrome or learning disability; a command of spoken English that was inadequate for engaging in therapy; and currently having individual CBT. All patients provided written informed consent.

### Randomisation and masking

We randomly assigned (1:1) eligible patients, after a baseline assessment, to either six sessions of CBT worry-reduction intervention done over 8 weeks added to standard care (the CBT intervention group), or to standard care alone (the control group). We used a web-based randomisation system, written by the Oxford Clinical Trials Unit for Mental Illness with a stratified randomisation procedure including four strata and a randomly permuted block procedure with variable block sizes. We did the stratification on the basis of centre and level of worry (defined as moderate when the PSWQ worry score was 44–62, and high when the score was ≥63).

The assessors were masked to patients' treatment allocations, but all patients were informed of their allocation by a trial therapist. Precautionary strategies included thinking about the best room to use and diary arrangements; patients being reminded by the assessors not to talk about allocation; and, after the initial assessment, the assessors did not look at clinical notes. If an allocation was revealed to the assessor, then remasking occurred, by use of another rater, which happened 11 times. However, if an allocation was revealed during an assessment session then these ratings were used: two 8-week assessments (both with the intervention) and four 24-week assessments (three with the intervention) were done unmasked.

### Procedures

We aimed to provide the CBT worry-reduction intervention in six sessions over 8 weeks. Each session lasted roughly an hour and took place in NHS clinics or at patients' homes. Therapy was delivered individually. Before therapy began the clinician met the patient for an initial introduction and assessment. The assessments of outcome measures were completed at 0 weeks (baseline), 8 weeks (end of therapy), and at 24 weeks (follow-up). Three graduate psychologists (EČ, GW, and KS) did the enrolment and assessments.

The highly detailed intervention is designed to provide clear and simple messages for patients to take into their day-to-day lives. We wrote a set of six session booklets, shared by the patient and therapist. The worry reduction strategies included have been shown to be effective at reducing worry and do not challenge the delusion itself. The main techniques were psychoeducation about worry, identification and reviewing of positive and negative beliefs about worry, increasing awareness of the initiation of worry and individual triggers, use of worry periods, planning activity at times of worry (which could include relaxation), and learning to let go of worry. We formulated a so-called worry cycle early in the intervention: feeling under threat leads to activation of positive beliefs about worry and hence engagement in this thinking style, resulting in dwelling on the worst outcomes and an increase in the initial feelings of threat. The worry cycle was discussed in relation to a recent bout of worry by the patient. Tasks were set between sessions—eg, implementation of worry periods. Whenever patients agreed, the trial therapists telephoned or texted them between sessions, to encourage them to try the new strategies. We helped patients to learn that they had understandable positive beliefs about worry (eg, that worry kept them safe) that meant that they engaged with this thinking style. They were helped to see the skewed view that worry provides and how it exacerbates fears. The two main practical techniques to reduce worry were then introduced: the use of worry periods (confining worry to about a 20 min set period each day) and planning of activities at peak worry times. Worry periods were implemented flexibly. For example, most patients set up one worry period a day, but they could choose to have two worry periods a day or, in severe instances, patients instead aimed for a worry-free period. Ideally, the worry period was then substituted with a problem-solving period. Our general approach and techniques are also described in a treatment book.^18^

Three clinical psychologists provided therapy (KP, JC, and HM), and were supervised each week by DF and HS. One of the therapists provided the intervention for all participants in Oxford (KP). The trial began with another therapist (JC) providing all therapy in Southampton, although in the latter part of the trial a third therapist took over (HM). We recorded therapy sessions when patients gave permission. To assess treatment fidelity, 12 recordings, chosen randomly, were rated on the Cognitive Therapy Scale—Revised (CTSR)^19^ by an independent clinical psychologist who was skilled in CBT for psychosis. All chosen recordings were rated as providing at least satisfactory cognitive therapy (ie, a mean score of at least 3).

Standard care was delivered according to national and local service protocols and guidelines. This usually consists of prescription antipsychotic drugs, visits from a community mental health worker, and regular outpatient appointments with a psychiatrist. It was recorded with the Client Service Receipt Inventory.^20^

### Outcomes

The pre-specified primary outcome measures were levels of worry assessed by the PSWQ^21^ and levels of persecutory delusions assessed by the PSYRATS-delusions scale.^16^ High scores on these scales indicate high levels of worry and delusions, respectively. Secondary outcome measures were delusion distress measured by the PSYRATS-distress scale; total psychiatric symptoms measured by the Positive and Negative Syndromes Scale (PANSS);^22^ paranoia measured by the Green et al Paranoid Thoughts Scale (GPTS);^23^ rumination measured by the Perseverative Thinking Questionnaire (PTQ);^24^ an adapted service user-led measure of patient outcomes (CHOICE)^25^ assessing—eg, self-confidence, having coping strategies, and a sense of being in control; and wellbeing measured by the Warwick-Edinburgh Mental Wellbeing Scale (WEMWBS).^26^ High scores on these scales indicate delusion distress, higher overall levels of psychiatric symptoms, paranoia, rumination, patient satisfaction, and psychological wellbeing. We tested interrater reliability for the two interviewer-rated assessments, with two-way mixed, one-measure intraclass correlations (ICC).

At baseline, to examine additional moderators of outcome, participants completed assessments of intellectual functioning (the Wechsler Adult Intelligence Scale [WAIS]),^27^ illicit drug use (the Maudsley Addiction Profile),^28^ illness and treatment representations,^29^ probabilistic reasoning,^30^ and working memory (appendix).^31^, ^32^

During the trial, we recorded any adverse event that came to our attention. We also checked medical notes at the end of the trial for the following events prespecified as adverse: all deaths, suicide attempts, serious violent incidents, admissions to secure units, and formal complaints about therapy.

### Statistical analysis

Our target sample size was 150 patients, split equally between the two centres. We wanted to detect moderate or large effects. A simple two-tailed t-test with 60 people per group would provide 90% power to detect an effect size of 0·60 at a significance level of 0·05, and would have 80% power to detect an effect size of 0·52. In practice, further power would be gained by use of multiple regression. Therefore, conservatively allowing for a 20% dropout, 150 people would need to be recruited to enable full data to be obtained from 120 participants.

We did all main analyses at the end of the last follow-up assessments at week 24 (ie, we did not do any interim analyses) with Stata version 13,^33^ in the intention-to-treat population, with due consideration being given to potential biases arising from loss to follow-up. Random or mixed effects models (with Stata's xtreg command) were fitted to the repeated measures to estimate treatment effects for outcomes, controlling for stratum (treatment centre crossed by the initial level of worry; ie, moderate or high), and the corresponding baseline assessment for the outcome being investigated. To find out whether the intervention effects differed at 8 weeks compared with 24 weeks (ie, whether effects were maintained), we also tested treatment by follow-up time interactions; this analysis tested whether differences in the intention-to-treat effects at the two follow-up times were significant. We allowed for the presence of missing outcome data under the assumption that the data were missing at random.^34^ We calculated standard effect sizes (Cohen's d) by dividing the estimated treatment effects by the pooled SD at follow-up.

We did all mediation analyses using the structural equation modelling package Mplus Version 7 (appendix).^35^ Our mediation analysis strategy was similar to that advocated by Baron and Kenny^36^—ie, we tested for intervention effects on the outcome (delusions) and on the proposed mediator, then fitted a full model to estimate the direct and indirect effects of the intervention on outcome—but with statistical models that account for the repeated measures of both mediator and outcome (ie, a parallel process model),^37^ acknowledge that confounding of the effect of mediator on outcome is probable,^38^ and allow for the fact that the mediator and outcome are subject to substantial measurement error.^39^

We used data from both treatment groups in these analyses—essentially assessing what proportion of the intention-to-treat effect of the worry intervention on delusions is attributed to its effect on worry. All statistical testing was two-tailed.

Level of worry was assumed to be the mediator and severity of paranoia the final outcome (rather than vice versa)—primarily motivated by the fact that the intervention was specifically targeted on worry as the mechanism of change. The parameters of the chosen model were then estimated assuming the underlying validity of the model.

We started with two simple measurement or factor analysis models—the first for worry and the second for delusions. In each case, the loadings for 8 and 24 weeks were constrained to be 1, the intercept term for each timepoint was constrained to be 0, and the variances of the measurement errors were equal for the two timepoints. We assumed that measurements at the follow-up times were parallel measures of a stable underlying latent variable.^35^ The measurement errors for worry and delusions were correlated at 8 weeks and 24 weeks.

We estimated the effects of the intervention on the worry outcome factor, the delusions outcome factor, and the effect of the worry factor on the outcome factor, allowing for a direct effect of the intervention on the outcome. In practice, the worry and delusions outcome factors were assessed in a joint structural equation model, allowing for the residual (ie, not accounted for by the intervention and baseline covariates) variation in worry and delusion to be correlated (as would be expected if mediation were present). For the effect of worry on the outcome, we jointly modelled the effect of the intervention on worry and the effect of worry and the intervention on outcome (this time not allowing the residuals to be correlated).

We allowed for confounding mainly by inclusion of the baseline values of both worry and delusions (in addition to the stratifying factors) in all the structural equation model analyses. In the intention-to-treat analyses there was no difficulty of confounding and the covariates were included to strengthen precision. In the mediation analysis we looked at a non-randomised comparison (neither mediator or outcome are under the direct control of the investigator), and confounding might therefore be present. A major source of such confounding is likely to be the correlation between the baseline values of worry and delusions (estimated here to be 0·51).

The main mediation analysis model was essentially equivalent to an analysis of covariance model for the effects on the intervention on the latent outcome common to 8 weeks and 24 weeks outcome, conditioning on the corresponding latent mediator and baseline covariates. An alternative approach to the analysis might have been through the use of latent change score models^37^—but, if no changes were shown in either mediator or outcomes between 8–24 weeks follow-up, the results of fitting an appropriately parameterised and constrained latent change model would yield identical results (ie, identical goodness-of-fit indices and identical parameter estimates for the direct and indirect effects of the intervention; appendix). A data monitoring and ethics committee oversaw our study. This trial is registered with the ISRCTN Registry, number ISRCTN23197625.

### Role of the funding source

The funder of the study reviewed the application for the trial and monitored the progress of trial milestones (eg, recruitment). The funder had no role in study design, data collection, data analysis, data interpretation, or writing of the report. The corresponding author had full access to all the data in the study and had final responsibility for the decision to submit for publication.

## Results

**Figure 1:**
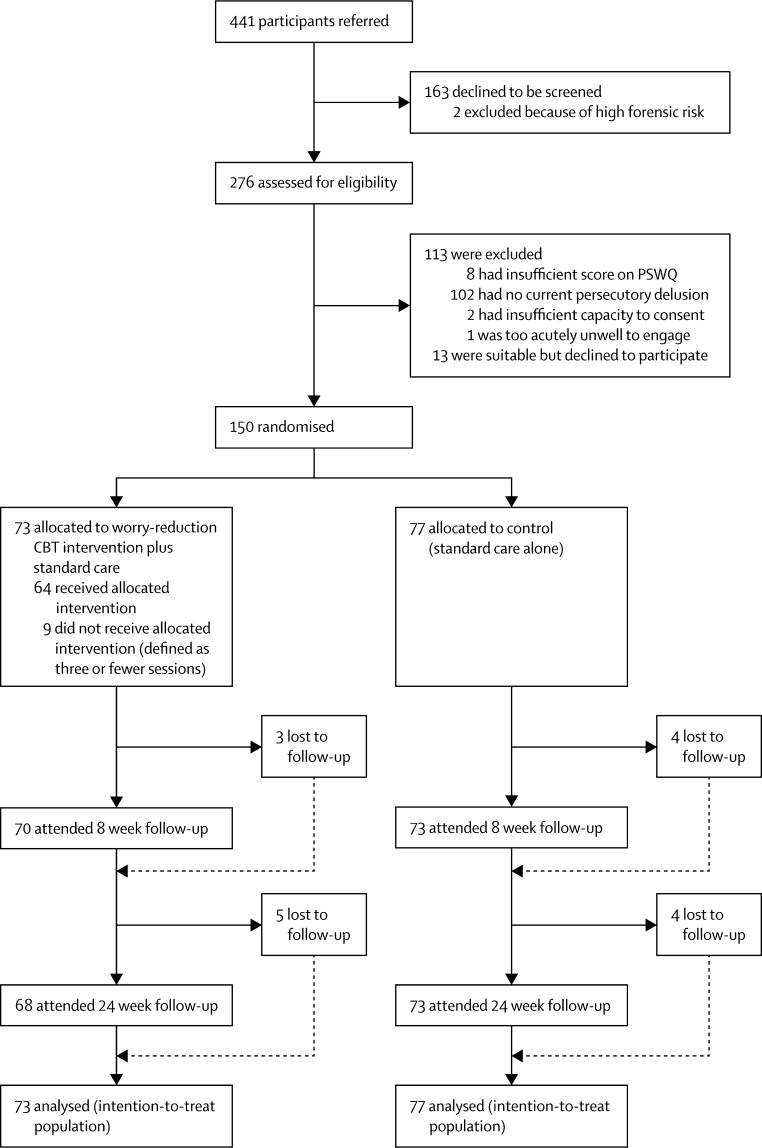
Trial profile PSWQ=Penn State Worry Questionnaire. CBT=cognitive behavioural therapy.

Between Nov 1, 2011, to Sept 9, 2013, with the last assessments completed on March 10, 2014, we assessed 276 participants, of whom 150 were eligible, gave imformed consent, and were randomly assigned to either the CBT intervention group (n=73) or to the control group (n=77; figure 1). As with other studies of persistent psychotic occurrences, both groups had a slightly higher preponderance of men than women, the mean age was around 40 years, most were unemployed, and the main psychiatric diagnosis was schizophrenia. All but nine patients were taking antipsychotic drugs (one in the CBT group, eight in the control group). Most patients had been in contact with mental health services for many years (table 1).

The mean number of sessions received was 5·5 (SD 1·8); 51 patients attended six sessions. In the interest of flexibility, for a few patients the intervention was provided in seven (n=7) or eight sessions (n=2) during the 8 week period. Two patients attended no therapy sessions. The remainder of the patients attended one (n=5), two (n=1), three (n=1), four (n=3), or five (n=1) sessions. Panel 1 shows patient comments about the intervention. An analysis of the effects of increasing compliance with therapy had been proposed in the published trial protocol^14^ but, in the event, compliance with the allocated intervention was so high that such an analysis was deemed unnecessary.

The therapist in Oxford provided the intervention to 37 participants. The two therapists in Southampton provided the intervention to 22 and 14 participants, respectively. The number of trial participants that can be used as controls for each of these three therapists was 37 for Oxford, and 23 and 13 for Southampton. In the sensitivity analyses allowing for therapist effects described in the section on mediation, trial participants were, in effect, stratified by therapist instead of centre. For inter-rater reliability tests, when rater 1 attended 23 assessments with rater 2, their reliability ratings were PSYRATS total ICC=0·99, PANSS total ICC=0·83. Rater 1 attended 18 assessments with rater 3 and their reliability ratings were PSYRATS total ICC=0·98, PANSS total ICC=0·75.

When compared with standard care alone, the CBT intervention led to a significant reduction in levels of worry (table 2). The estimated mean difference in PSWQ scores at 8 weeks between the CBT-intervention group and the control group was 6·35 (SE 1·56; 95% CI 3·30–9·40; p<0·001). Persecutory delusions were also reduced in the CBT-intervention group compared with the control group; the estimated mean difference in PSYRATS scores at 8 weeks in the intervention group compared with the standard care group was 2·08 (SE 0·73; 95% CI 0·64–3·51; p=0·005). The mean treatment by follow-up time (8 and 24 weeks) interactions were estimated to be −2·43 PSWQ (SE 1·57; p=0·121) and 0·86 PSYRATS (SE 0·68; p=0·205), suggesting that at 24 weeks, the treatment effects were smaller for PSWQ, but larger for PSYRATS. However, neither of these interactions were significant and the statistical models were refined to estimate treatment effects (ie, differences in average outcome between the two randomised groups) that were assumed to be common to both follow-up times. The resulting treatment-effect estimates were 5·15 (SE 1·35; 95% CI 2·50–7·79; p<0·001; Cohen's d=0·47) and 2·50 (SE 0·65; 95% CI 1·22–3·78; p<0·001; Cohen's d=0·49). No substantial temporal trends in the mediator or the outcome between 8 and 24 weeks were noted, substantially simplifying the statistical models needed for the analysis of the associations between changes in the mediator and the corresponding changes in clinical outcome.

Significant improvements were noted with the CBT treatment for all the secondary outcome measures. There were no significant treatment by follow-up time interactions (ie, intention-to-treat effects did not significantly differ between 8 weeks and 24 weeks), and therefore treatment estimates common to both follow-ups were made. Compared with standard care alone, CBT intervention reduced mean PSYRATS distress scores (0·85, SE 0·25, p=0·001, Cohen's d=0·41), PANSS psychiatric symptom scores (6·16, SE 1·69, p<0·001, Cohen's d=0·42), paranoia GPTS scores (14·68, SE 4·18, p<0·001, Cohen's d=0·45), and rumination PTQ scores (3·51, SE 1·43, p=0·014, Cohen's d=0·32). We noted improvements in the intervention group versus standard care group in psychological wellbeing WEMWBS scores (2·40, SE 1·11, p=0·03, Cohen's d=0·23) and patient chosen outcomes CHOICE scores (10·45, SE 2·42, p<0·001, Cohen's d=0·52).

Treatment effects were not moderated by centre, therapist, level of worry or delusions, intellectual functioning, illicit drug use, illness perceptions, reasoning, or working memory (p>0·05).

**Figure 2:**
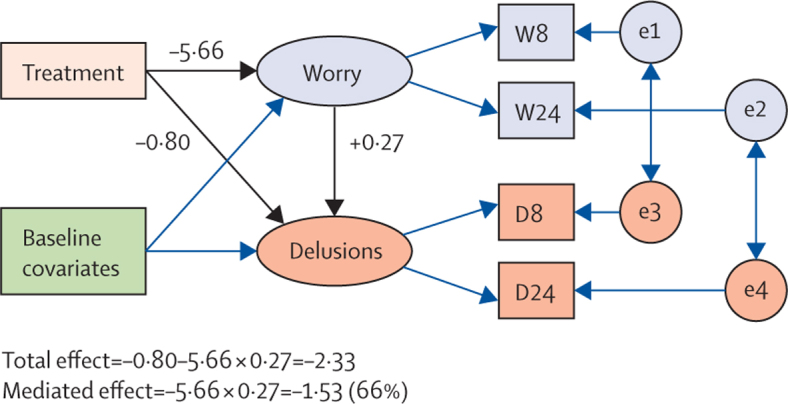
Mediation analysis Rectangles or squares represent measured variables. Ellipses or circles represent latent variables (including random errors or residuals). Single headed arrows represent predisposing effects; bold arrows represent main ones of interest. Double-headed arrows represent correlations. W8=worry measures at 8 weeks. W24=worry measures at 24 weeks. D8=delusion measures at 8 weeks. D24=delusion measures at 24 weeks. e1 and e2=random residuals (worry). e3 and e4=random residuals (delusions).

Figure 2 provides an overview of the mediation analysis. The CBT intervention reduced the worry factor by a mean of 5·66 (SE 1·32, 95% CI 3·08–8·24; p<0·001) and the delusions factor by a mean of 2·33 units (SE 0·64, 95% CI 1·08–3·58; p<0·001). The intervention directly reduced the delusion factor by a mean of 0·80 (SE 0·65, 95% CI −0·70 to 2·07; p=0·214). Each unit reduction in the worry factor produced a 0·27 change in the delusions factor (SE 0·06, 95% CI 0·15–0·39; p<0·001). The estimated indirect (mediated) effect of the intervention on the delusions factor was a reduction of 1·53 (SE 0·49, 95% CI 0·57–2·48; p=0·002). The proportion of the effect of the intervention on outcome (delusions) that is mediated by changes in worry is therefore 1·53/2·33=66%. The structural equation model fitted the data as shown by a χ^2^ score of 20·03 with 17 degrees of freedom (p=0·273), a root mean square error of approximation of 0·035, and comparative fit index of 0·992.

One concern about the validity of the estimate of effect of change in worry on change in delusions came from the possibility of confounding arising from differential therapist effects. However, when we used therapist identity as a covariate in the models instead of treatment centre (but not including a worry stratum by therapist interaction), the estimated effect of worry on delusions was unchanged: 0·27 (SE 0·06). The further addition of the therapists by treatment interactions (acknowledging that differences might occur in the effectiveness of the therapists) as covariates produced identical results.

The standard care provided for each group was similar between groups (table 3). Data for the number of days in hospital is skewed for the CBT treatment group, because one patient was in hospital for 2 years before entering the trial, although they were discharged 3 months into the trial.

Two patients did not give us permission to check medical notes at the end of the trial. No deaths, admissions to secure units, or formal complaints about therapy occurred during the trial. There were six suicide attempts (two in the treatment group, four in the control group) and two serious violent incidents (one in each allocation group). None of the adverse events were related to therapy or the assessments.

## Discussion

The results of the planned analysis were entirely consistent with the inference that treating worry in patients with persecutory delusions leads to reductions in delusions. With the psychological treatment, patients also had several other important outcomes, such as a reduction in overall levels of psychiatric symptoms and general levels of paranoid thinking, and an improvement in psychological wellbeing (Panel 1, Panel 2).

Traditionally, a fundamental divide has been made between neurosis and psychosis. Worry was studied and treated in emotional disorders, but not in psychosis. Ironically, our WIT study, to our knowledge,^40^ is the largest trial so far of a psychological treatment for patients with clinical worry, but it was undertaken in patients with diagnoses of psychosis. Our study was based on a theoretical understanding of the role of worry in delusions, empirical studies that suggested an important link, and the results of a promising pilot study.^12^ The group given treatment had severe persecutory delusions that had not responded sufficiently to other treatments. The main outcomes were very clear. A brief cognitive behavioural intervention for worry, compared with treatment as usual, led to significant reductions in both worry and the persecutory delusions.

Patients liked the focus on worry, seen in the high uptake of the therapy sessions. They agreed that they had this problem; nonetheless, by reducing their preoccupation with threat and increasing activity levels, the persecutory delusions were implicitly challenged. Some patients, by being more active with the goal of dealing with worry, learned that they were safer outside than they had feared. Only eight patients with persecutory delusions were excluded from entering the trial on the basis of reporting insufficient worry. The intervention was deliberately highly detailed to help with later dissemination. The length of therapy was remarkably short to achieve such change in long-standing delusional beliefs. Agreeing to six sessions help both the patient and therapist to initiate active techniques early, and keeps therapy precisely focused. Nevertheless, we do not envisage that the worry intervention is sufficient psychological help for these patients; they still had significant levels of worry and paranoia at 24 weeks follow-up—therefore the benefits need to be enhanced and maintained over longer periods. We are now beginning to test the worry intervention in combination with modular interventions targeting other key causal factors, such as sleep disturbance, reasoning biases, and low self-esteem.^1^ The intervention will probably have wider applicability—eg, to patients at high risk of psychosis,^41^ patients at first episode of psychosis, and to patients with other disorders for which worry is a putative contributory cause.

Our investigation had three main limitations. We did not include a condition to control for therapist contact; however, this was because the most important aspect in this explanatory study was to show a change in the putative causal factor—worry—so that any effects on delusions could be assessed. In this mechanistic trial, change in the worry thinking style needed to be established, not the components of therapy that might achieve this. For example, although we think it highly unlikely that befriending or supportive counselling would have such persistent effects on worry and delusions, this possibility will have to be tested specifically in this patient group. Importantly, substantial limitations exist in what can be established definitively with regard to mediation. In our investigation, we could not rule out the possibility that the intervention has merely created non-specific change in a range of outcomes; against this possibility, the largest effect sizes for psychiatric symptoms were for the two that were targeted—worry and persecutory delusions. These positive effects of six sessions of therapy persisted at 6 months. Worry is a transdiagnostic process, and therefore many benefits could probably be gained by reducing worry. (The control group showed some improvement, which is typical with the monitoring that occurs during a clinical trial.) We did not aim to measure temporal associations between changes in worry and changes in the delusion. Although the worry style was the target of intervention, and not the content of the delusions, the statistical models cannot definitively rule out reverse causation—indeed, a reciprocal association between worry and paranoia is plausible—or possible hidden confounding (particularly those arising from experiences and life-events that occurred during the trial but were assumed to be unrelated to the trial intervention). Overall, we note the advice of Bullock and colleagues^42^ “to think of mediation analysis as a cumulative enterprise”. The study cannot definitively show mediation, but the results are consistent with reports in the theoretical and empirical scientific literature and the focus of the intervention techniques. Finally, follow-up was only roughly 4–6 months after the end of treatment, though we regard this time as appropriate for such a short intervention. In clinical practice, booster sessions should be added. We hope to see further clinical trials that focus specifically on persecutory delusions.

## Figures and Tables

**Table 1:** Baseline characteristics of the intention-to-treat population

		**CBT intervention group (n=73)**	**Control group (n=77)**
Age (years)		40·9 (10·5)	42·1 (12·2)
Sex			
	Male	42 (58%)	44 (57%)
	Female	31 (42%)	33 (43%)
Ethnic origin			
	White	68 (93%)	69 (89%)
	Black	1 (1%)	0 (0%)
	Chinese	0 (0%)	2 (3%)
	Indian	0 (0%)	3 (4%)
	Other	4 (6%)	3 (4%)
Employment status			
	Unemployed	55 (75%)	51 (66%)
	Part-time employed	8 (12%)	6 (7%)
	Full-time employed	3 (4%)	10 (13%)
	Self employed	1 (1%)	2 (3%)
	Retired	2 (3%)	6 (8%)
	Student	1 (1%)	2 (3%)
	Housewife or husband	3 (4%)	0 (0%)
Intelligence quotient		100·3 (19·0)	101·8 (18·2)
Diagnosis			
	Schizophrenia	58 (79%)	53 (69%)
	Schizoaffective disorder	5 (7%)	6 (7%)
	Delusional disorder	4 (5%)	6 (7%)
	Psychosis NOS	6 (8%)	12 (16%)
Outpatient		71 (97%)	76 (99%)
Inpatient		2 (3%)	1 (1%)
Inpatient admission in previous 6 months		10 (14%)	8 (10%)
Chlorpromazine-equivalent dose of antipsychotic drug (mg/day)		523·2 (394·3)	475·5 (420·6)
Time in contact with services			
	<1 year	5 (8%)	7 (9%)
	1–5 years	12 (16%)	17 (22%)
	6–10 years	16 (22%)	12 (16%)
	11–20 years	18 (25%)	26 (34%)
	>20 years	21 (29%)	15 (19%)

Data are n (%) or mean (SD). NOS=not otherwise specified.

**Table 2:** Primary and secondary outcome measures

		**CBT intervention group**		**Control group**	
		n	Mean (SD)	n	Mean (SD)
**Primary measures**					
Worry (PSWQ)					
	0 weeks	73	64·8 (8·6)	77	64·5 (9·5)
	8 weeks	70	54·8 (10·5)	73	61·0 (12·2)
	24 weeks	68	56·1 (9·7)	73	59·8 (11·0)
Delusion (PSYRATS-delusion)					
	0 weeks	73	18·7 (3·0)	77	18·0 (3·0)
	8 weeks	70	14·3 (4·8)	73	15·9 (5·1)
	24 weeks	68	13·6 (5·6)	72	16·4 (4·8)
**Secondary measures**					
Delusion distress (PSYRATS-distress)					
	0 weeks	73	6·4 (1·4)	77	6·5 (1·3)
	8 weeks	70	5·1 (1·9)	73	5·8 (2·1)
	24 weeks	68	5·0 (2·2)	72	6·1 (1·8)
Total symptoms (PANSS)					
	0 weeks	73	82·0 (13·6)	76	79·0 (13·5)
	8 weeks	69	70·7 (12·4)	73	75·3 (16·0)
	24 weeks	68	71·5 (15·4)	71	76·3 (16·7)
Paranoia (GPTS)					
	0 weeks	73	115·9 (27·3)	77	110·8 (27·8)
	8 weeks	70	90·0 (32·2)	73	102·3 (31·7)
	24 weeks	67	92·5 (32·7)	73	105·6 (32·4)
Rumination (PTQ)					
	0 weeks	70	44·3 (9·7)	72	44·9 (9·8)
	8 weeks	68	37·7 (9·7)	70	41·0 (11·7)
	24 weeks	64	37·3 (10·5)	71	42·7 (10·6)
Patient outcomes (CHOICE)					
	0 weeks	71	49·4 (17·3)	75	49·5 (18·5)
	8 weeks	67	64·4 (17·1)	69	51·7 (21·1)
	24 weeks	66	61·6 (21·4)	70	52·5 (22·4)
Wellbeing (WEMWBS)					
	0 weeks	73	36·4 (9·6)	77	34·5 (9·2)
	8 weeks	68	41·5 (9·1)	73	36·5 (11·3)
	24 weeks	67	40·2 (10·8)	73	36·6 (10·5)

CBT=cognitive behavioural therapy. PSWQ=Penn State Worry Questionnaire. PSYRATS=Psychotic Symptoms Rating Scale. PANSS=Positive and Negative Syndromes Scale. GPTS=Green et al Paranoid Thoughts Scale. PTQ=Perseverative Thinking Questionnaire. CHOICE=CHoice of Outcome In Cbt for psychosEs. WEMWBS=Warwick-Edinburgh Mental Wellbeing Scale.

**Table 3:** Standard care provided in the CBT intervention group and the control group

	**CBT intervention group**		**Control group**	
	n	Mean (SD)	n	Mean (SD)
**6 months before the trial**				
Number of days in hospital	73	7·4 (26·8)	77	2.·8 (9·5)
Meetings with psychiatrist	72	2·4 (3·9)	77	2·8 (4·2)
Meetings with community psychiatric nurse	72	12·3 (9·9)	76	10·5 (10·1)
Meetings with counsellor or therapist	72	1·5 (6·2)	77	1·1 (4·7)
Visits to day-care centre	72	0·8 (4·3)	77	1·7 (10·6)
GP meetings	73	3·8 (4·8)	77	2·6 (3·2)
**6 months during the trial**				
Number of days in hospital	73	3·5 (15·0)	77	0·2 (1·6)
Meetings with psychiatrist	65	1·6 (1·9)	71	1·8 (2·2)
Meetings with community psychiatric nurse	65	11·2 (11·3)	71	9·2 (13·9)
Meetings with counsellor or therapist (outside of the trial)	61	1·0 (3·6)	66	1·1 (3·4)
Visits to day-care centre	65	0·4 (2·6)	71	1·0 (6·3)
GP meetings	65	2·6 (2·6)	71	2·6 (2·5)

Data are n, mean (SD). CBT=cognitive behavioural therapy. GP=general practitioner.

## Panel 1 Patient comments on the intervention

**Patient 1**

“The discussions about preventing worry and reducing worry were extremely helpful to me. It made me see my worry as something real. The breakthrough was that I was able to, with the help of my psychologist, come up with a strategy—that is, when worry [was] gripping me I would say “‘excuse me worry, while I do…’” or “‘excuse me worry, I need to interrupt you because….’” I sometimes worry about people trying to harm me but now I can interrupt my worry and do something else. I challenge myself to do this because I know it works for me.”

**Patient 2**

“I had no confidence in who I was and felt I avoided everyone because of my thoughts and being in company was really frightening. I found the therapy challenging and sometimes very difficult. But it was eye-opening as I didn't realise how much I worried and where the worry was coming from. To see it on paper made it more straightforward and made my life more clear. I do feel that I now try to take time out, whether that's a cup of tea or going to the shops. Just doing things that I actually enjoy doing and building on them as I was so wrapped up in anxiety I was lost. I am more relaxed at certain times of the day where I was once completely stressed. I still find it hard around people but I feel I can still build on the skills you gave me and if it's slightly better, that's good.”

**Patient 3**

“The therapy was very rewarding. There wasn't anything I didn't like. I needed that kind of therapy at the time because if I didn't have that therapy at that time, I wouldn't be here. It was therapeutic talking about things. I listened to what you had to say and wrote down how I felt. I also tried relaxing to the tape and I ignored people when they were horrible to me. It was hard becoming disciplined but we worked as a team, that's what I liked about it. You don't get nowhere in this world if you don't work as a team. I was having a hard time and you was doing your best to stop me having a hard time. That's what I call team work; I couldn't have been able to do it by myself, no way. I thought a lot about what I thought the therapy did—it decreased my worrying but in other ways it built my confidence.”

## Panel 2 Research in context

**Systematic review**

We searched the ISRCTN trial registry and the PubMed database with the search terms “worry”, “delusions”, “persecutory”, “paranoia”, and “schizophrenia” without date restrictions, for English-language publications of randomised controlled trials investigating the treatment of worry in patients with persecutory delusions. Other than our pilot investigation^12^ there were no other such clinical trials in the medical literature. We also examined published meta-analyses on standard cognitive behavioural therapy (CBT) for persistent delusions or hallucinations, or both.^43^

**Interpretation**

After our pilot study,^12^ we have shown for the first time that treating worry leads to reductions in persecutory delusions. This is new for the evidence-base and consistent with (though not definitive for) a role for worry as a contributory cause of paranoia. The effect size is similar to standard CBT for persistent psychotic occurrences, according to meta-analyses.^43^ Standard CBT for psychosis does not include standard worry-intervention techniques, but our intervention is much briefer. The trial shows the promise of taking a focused theoretically driven approach to the treatment of psychosis.
