# Supplementary material for: Effects of cognitive behaviour therapy for worry on persecutory delusions in patients with psychosis (WIT): a parallel, single-blind, randomised controlled trial with a mediation analysis
Source: Lancet Psychiatry. 2015 Mar 31;2(4):305–13. doi: 10.1016/S2215-0366(15)00039-5 (PMC4698664; doi:10.1016/S2215-0366(15)00039-5)
Supplement: Supplementary appendix [file mmc1.pdf]

THE LANCET Psychiatry

Supplementary appendix

This appendix formed part of the original submission and has been peer reviewed. We post it as supplied by the authors.

Supplement to: Freeman D, Dunn G, Startup H, et al. Effects of cognitive behaviour therapy for worry on persecutory delusions in patients with psychosis (WIT): a parallel, single-blind, randomised controlled trial with a mediation analysis. *Lancet Psychiatry* 2015; published online March 4. http://dx.doi.org/10.1016/S2215-0366(15)00039-5.

Mplus Mediation Model for the Worry Intervention Trial (WIT)

(a) Model used for the main mediation analyses

Important components of the output highlighted.

Input instructions

```
Title:
  Stata2Mplus conversion for WiTmediation2.dta
  List of variables converted shown below

  Centre :
    0: Oxford
    1: Southampton
  Worryscorestrata :
    0: Moderate worry score
    1: High worry score
  WorryMainMeasure_Baseline :
  WorryMainMeasure_8WEEK :
  WorryMeasure_24WEEK :
  PSYRATS_total_Baseline :
  PSYRATS_total_8WEEK :
  PSYRATS_total_24WEEK :
  treat :
  Interact :

Data:
  File is WiTmediation2.dat ;
Variable:
  Names are
     Centre Wstratum Worry0 Worry8 Worry24
     PSY0 PSY8 PSY24
     treat Interact ID;
  Usevariables=Centre Wstratum Worry0 Worry8 Worry24
     psy0 psy8 psy24 treat interact;
  Missing are all (-9999) ;
Analysis:
  Type = general;
  Iterations=10000;

Model:
worryfac by worry8@1 worry24@1;
worry8 worry24 (1);
[worry8@0 worry24@0];
 worry8 with psy8;
 worry24 with psy24;

psyfac by psy8@1 psy24@1;
psy8 psy24 (2);
[psy8@0 psy24@0];

worryfac on treat (A)
Centre Wstratum Interact worry0 psy0;

psyfac on worryfac (B)
treat (C)
```

```
Centre Wstratum Interact worry0 psy0;

worryfac with psyfac@0;
worryfac psyfac;
[worryfac psyfac];

Model indirect:
psyfac ind treat ;

! MODEL CONSTRAINT:
!       new (D propmed);
!       D=A*B;
!       propmed=D/(D + C);

Output:
cinterval;
```

Model fit information

Number of Free Parameters 21

Loglikelihood

H0 Value -1766.801
H1 Value -1756.784

Information Criteria

Akaike (AIC) 3575.601
Bayesian (BIC) 3637.821
Sample-Size Adjusted BIC 3571.374
(n* = (n + 2) / 24)

Chi-Square Test of Model Fit

Value 20.034
Degrees of Freedom 17
P-Value 0.2725

RMSEA (Root Mean Square Error Of Approximation)

Estimate 0.035
90 Percent C.I. 0.000 0.087
Probability RMSEA <= .05 0.621

CFI/TLI

CFI 0.992
TLI 0.985

Chi-Square Test of Model Fit for the Baseline Model

Value 390.351
Degrees of Freedom 30
P-Value 0.0000

SRMR (Standardized Root Mean Square Residual)

Value 0.065

Model results

	Estimate	S.E.	Est./S.E.	Two-Tailed P-Value
WORRYFAC BY				
WORRY8	1.000	0.000	999.000	999.000
WORRY24	1.000	0.000	999.000	999.000
PSYFAC BY				
PSY8	1.000	0.000	999.000	999.000
PSY24	1.000	0.000	999.000	999.000
PSYFAC ON				
WORRYFAC	0.270	0.059	4.561	0.000
WORRYFAC ON				
TREAT	-5.660	1.318	-4.295	0.000
CENTRE	-3.725	2.021	-1.844	0.065
WSTRATUM	-5.354	2.589	-2.068	0.039
INTERACT	8.084	2.660	3.039	0.002
WORRY0	0.601	0.130	4.616	0.000
PSY0	0.358	0.260	1.381	0.167
PSYFAC ON				
TREAT	-0.804	0.647	-1.242	0.214
CENTRE	0.833	0.879	0.947	0.343
WSTRATUM	-0.490	1.132	-0.432	0.666
INTERACT	-0.926	1.216	-0.762	0.446
WORRY0	-0.069	0.065	-1.052	0.293
PSY0	0.691	0.111	6.211	0.000
WORRYFAC WITH				
PSYFAC	0.000	0.000	999.000	999.000
WORRY8 WITH				
PSY8	10.896	2.241	4.862	0.000
WORRY24 WITH				
PSY24	3.663	2.307	1.588	0.112
Intercepts				
WORRY8	0.000	0.000	999.000	999.000
WORRY24	0.000	0.000	999.000	999.000
PSY8	0.000	0.000	999.000	999.000
PSY24	0.000	0.000	999.000	999.000
WORRYFAC	17.787	7.067	2.517	0.012
PSYFAC	-8.161	3.156	-2.586	0.010
Residual Variances				
WORRY8	44.842	5.351	8.381	0.000
WORRY24	44.842	5.351	8.381	0.000
PSY8	7.926	0.931	8.514	0.000
PSY24	7.926	0.931	8.514	0.000
WORRYFAC	38.989	7.664	5.088	0.000
PSYFAC	7.432	1.349	5.509	0.000

(b) Equivalent Latent Change Score Model

```
ANALYSIS:   ESTIMATOR = ML;
            INFORMATION = OBSERVED;
            ITERATIONS = 50000;
MODEL:

!driving variables: M
        Worry8 ON Worry0@1;
        Worry24 ON Worry0@1;
        Wchange BY Worry8@1 Worry24@1;

        Worry8*5 Worry24*5 (e1);

        [Worry8@0 Worry24@0];

        Wchange ON Worry0 (a1);

        Wchange*5;
        [Wchange*0];

!driving variable: Y
        PSY8 ON PSY0@1;
        PSY24 ON PSY0@1;
        Pchange BY PSY8@1 PSY24@1;

        PSY8*5 PSY24*5 (e2);
        [PSY8@0 PSY24@0];

        Pchange ON PSY0 (a2);

        Pchange*5 ;
        [Pchange*0];


!bivariate relationships

        Wchange ON PSY0 Worry0;

        Pchange ON Worry0 PSY0 ;

        Pchange ON Wchange (b7);

        Worry8 with PSY8*0.2; (c1)
        Worry24 with PSY24*0.2; (c1)

!group effects
        Wchange ON treat*0 (g1)
        centre wstratum interact;
        Pchange ON treat*0 (g3)
        centre wstratum interact;
        Wchange with Pchange@0;

      MODEL CONSTRAINT:
        NEW (IND DIR TEY);
        IND = b7*g1; !indirect effect of y1
        DIR = g3;    !direct effect of y1
        TEY = g3 + b7*g1;
```

Model fit information

Number of Free Parameters	21

Loglikelihood

H0 Value	-1766.801
H1 Value	-1756.784

Information Criteria

Akaike (AIC)	3575.601
Bayesian (BIC)	3637.821
Sample-Size Adjusted BIC (n* = (n + 2) / 24)	3571.374

Chi-Square Test of Model Fit

Value	20.034
Degrees of Freedom	17
P-Value	0.2725

RMSEA (Root Mean Square Error Of Approximation)

Estimate	0.035	
90 Percent C.I.	0.000	0.087
Probability RMSEA <= .05		0.621

CFI/TLI

CFI	0.992
TLI	0.985

Chi-Square Test of Model Fit for the Baseline Model

Value	390.351
Degrees of Freedom	30
P-Value	0.0000

SRMR (Standardized Root Mean Square Residual)

Value	0.065

Model results

	Estimate	S.E.	Est./S.E.	Two-Tailed P-Value
WCHANGE BY				
WORRY8	1.000	0.000	999.000	999.000
WORRY24	1.000	0.000	999.000	999.000
PCHANGE BY				
PSY8	1.000	0.000	999.000	999.000
PSY24	1.000	0.000	999.000	999.000
PCHANGE ON				
WCHANGE	0.270	0.059	4.560	0.000
WCHANGE ON				
WORRY0	-0.399	0.130	-3.061	0.002
PSY0	0.358	0.260	1.381	0.167
TREAT	-5.660	1.318	-4.295	0.000
CENTRE	-3.725	2.021	-1.844	0.065
WSTRATUM	-5.354	2.589	-2.068	0.039
INTERACT	8.084	2.660	3.039	0.002
PCHANGE ON				
PSY0	-0.309	0.111	-2.780	0.005
WORRY0	0.201	0.060	3.366	0.001
TREAT	-0.803	0.647	-1.242	0.214
CENTRE	0.833	0.879	0.948	0.343
WSTRATUM	-0.489	1.132	-0.432	0.666
INTERACT	-0.926	1.216	-0.762	0.446
WORRY8 ON				
WORRY0	1.000	0.000	999.000	999.000
WORRY24 ON				
WORRY0	1.000	0.000	999.000	999.000
PSY8 ON				
PSY0	1.000	0.000	999.000	999.000
PSY24 ON				
PSY0	1.000	0.000	999.000	999.000
WCHANGE WITH				
PCHANGE	0.000	0.000	999.000	999.000
WORRY8 WITH				
PSY8	10.896	2.241	4.862	0.000
WORRY24 WITH				
PSY24	3.663	2.307	1.588	0.112
Intercepts				
WORRY8	0.000	0.000	999.000	999.000
WORRY24	0.000	0.000	999.000	999.000
PSY8	0.000	0.000	999.000	999.000
PSY24	0.000	0.000	999.000	999.000
WCHANGE	17.786	7.067	2.517	0.012
PCHANGE	-8.161	3.156	-2.586	0.010

Residual Variances

WORRY8	44.845	5.351	8.380	0.000
WORRY24	44.845	5.351	8.380	0.000
PSY8	7.926	0.931	8.514	0.000
PSY24	7.926	0.931	8.514	0.000
WCHANGE	38.989	7.664	5.087	0.000
PCHANGE	7.432	1.349	5.509	0.000

New/Additional Parameters

IND	-1.526	0.486	-3.142	0.002
DIR	-0.803	0.647	-1.242	0.214
TEY	-2.330	0.636	-3.664	0.000

Results of the moderation analysis

(Each potential moderator is included as an additional covariate before evaluating the possible treatment by moderator interaction.)

```
---------------------------------------------------------------------------------------------
--------------------------------------------
      name:  <unnamed>
       log:  C:\Users\MDEASGD2\Documents\Projects_Trials\Worry Intervention Trial\Worry Trial
Results\Moderators GD29March14.log
  log type:  text
 opened on:  29 Mar 2014, 14:21:54

. xi: regress WorryMainMeasure_8WEEK WorryMainMeasure_Baseline i.Centre*i.Worryscorestrata
i.treat
i.Centre          _ICentre_0-1        (naturally coded; _ICentre_0 omitted)
i.Worryscores~a   _IWorryscor_0-1     (naturally coded; _IWorryscor_0 omitted)
i.Cen~e*i.Wor~a   _ICenXWor_#_#       (coded as above)
i.treat           _Itreat_0-1         (naturally coded; _Itreat_0 omitted)

      Source |       SS       df       MS              Number of obs =     143
-------------+------------------------------           F(  5,   137) =   15.55
       Model |  7137.10754     5  1427.42151           Prob > F      =  0.0000
    Residual |  12576.6407   137  91.8002972           R-squared     =  0.3620
-------------+------------------------------           Adj R-squared =  0.3388
       Total |  19713.7483   142  138.829213           Root MSE      =  9.5812

------------------------------------------------------------------------------------------
   WorryMainMeasure_8WEEK |      Coef.   Std. Err.      t    P>|t|     [95% Conf. Interval]
--------------------------+---------------------------------------------------------------
WorryMainMeasure_Baseline |   .7170737   .1481978     4.84   0.000     .4240228    1.010125
               _ICentre_1 |  -5.017858    2.48309    -2.02   0.045    -9.927997   -.1077179
            _IWorryscor_1 |  -6.263745   3.167678    -1.98   0.050    -12.52761    .0001214
            _ICenXWor_1_1 |   9.870977   3.254168     3.03   0.003     3.436084    16.30587
                _Itreat_1 |  -6.347129   1.604075    -3.96   0.000    -9.519076   -3.175182
                    _cons |   17.80341   8.467442     2.10   0.037     1.059628     34.5472
------------------------------------------------------------------------------------------

. xi: regress WorryMainMeasure_8WEEK WorryMainMeasure_Baseline i.Centre*i.Worryscorestrata
i.treat*WAIS_WMDigitForward
i.Centre          _ICentre_0-1        (naturally coded; _ICentre_0 omitted)
i.Worryscores~a   _IWorryscor_0-1     (naturally coded; _IWorryscor_0 omitted)
i.Cen~e*i.Wor~a   _ICenXWor_#_#       (coded as above)
i.treat           _Itreat_0-1         (naturally coded; _Itreat_0 omitted)
i.treat*WAIS_~d   _ItreXWAIS__#       (coded as above)

      Source |       SS       df       MS              Number of obs =     120
-------------+------------------------------           F(  7,   112) =    9.41
       Model |  6314.40305     7  902.057578           Prob > F      =  0.0000
    Residual |  10741.3886   112  95.9052555           R-squared     =  0.3702
-------------+------------------------------           Adj R-squared =  0.3309
       Total |  17055.7917   119   143.32598           Root MSE      =  9.7931

------------------------------------------------------------------------------------------
   WorryMainMeasure_8WEEK |      Coef.   Std. Err.      t    P>|t|     [95% Conf. Interval]
--------------------------+---------------------------------------------------------------
WorryMainMeasure_Baseline |   .7407661   .1626941     4.55   0.000     .4184087    1.063124
               _ICentre_1 |  -3.910217    2.87373    -1.36   0.176    -9.604145    1.783711
            _IWorryscor_1 |  -7.560774   3.424847    -2.21   0.029    -14.34667   -.7748787
            _ICenXWor_1_1 |   8.672159   3.689063     2.35   0.020     1.362753    15.98157
                _Itreat_1 |   -4.73499   7.339495    -0.65   0.520    -19.27726    9.807278
      WAIS_WMDigitForward |  -.3925623   .5234837    -0.75   0.455    -1.429778    .6446535
            _ItreXWAIS__1 |  -.2448256   .7458686    -0.33   0.743    -1.722669    1.233018
                    _cons |   20.93953   11.23968     1.86   0.065    -1.330461    43.20952
------------------------------------------------------------------------------------------

. xi: regress WorryMainMeasure_8WEEK WorryMainMeasure_Baseline i.Centre*i.Worryscorestrata
i.treat*WAIS_WMDigitBack
i.Centre          _ICentre_0-1        (naturally coded; _ICentre_0 omitted)
i.Worryscores~a   _IWorryscor_0-1     (naturally coded; _IWorryscor_0 omitted)
i.Cen~e*i.Wor~a   _ICenXWor_#_#       (coded as above)
i.treat           _Itreat_0-1         (naturally coded; _Itreat_0 omitted)
i.treat*WAIS_~k   _ItreXWAIS__#       (coded as above)

      Source |       SS       df       MS              Number of obs =     119
-------------+------------------------------           F(  7,   111) =   10.55
```

```
       Model |  6788.41433     7  969.773476           Prob > F      =  0.0000
    Residual |  10199.4344   111  91.8867965           R-squared     =  0.3996
-------------+------------------------------           Adj R-squared =  0.3617
       Total |  16987.8487   118   143.96482           Root MSE      =  9.5858

------------------------------------------------------------------------------
   WorryMainMeasure_8WEEK |      Coef.   Std. Err.      t    P>|t|     [95% Conf. Interval]
--------------------------+---------------------------------------------------------------
WorryMainMeasure_Baseline |    .738846    .159108     4.64   0.000     .4235629    1.054129
               _ICentre_1 |  -4.141688   2.838673    -1.46   0.147    -9.766709    1.483332
            _IWorryscor_1 |  -7.928614   3.372201    -2.35   0.020    -14.61086   -1.246373
            _ICenXWor_1_1 |   9.765665   3.668966     2.66   0.009     2.495363    17.03597
                _Itreat_1 |  -3.536373   5.514778    -0.64   0.523    -14.46427    7.391528
         WAIS_WMDigitBack |  -.8760398   .5534741    -1.58   0.116    -1.972786    .2207061
            _ItreXWAIS__1 |  -.6588879   .8847844    -0.74   0.458    -2.412147    1.094371
                    _cons |   22.61717   10.22082     2.21   0.029     2.363928    42.87041
------------------------------------------------------------------------------

. xi: regress WorryMainMeasure_8WEEK WorryMainMeasure_Baseline i.Centre*i.Worryscorestrata
i.treat*WAIS_WMLetter
i.Centre          _ICentre_0-1        (naturally coded; _ICentre_0 omitted)
i.Worryscores~a   _IWorryscor_0-1     (naturally coded; _IWorryscor_0 omitted)
i.Cen~e*i.Wor~a   _ICenXWor_#_#       (coded as above)
i.treat           _Itreat_0-1         (naturally coded; _Itreat_0 omitted)
i.treat*WAIS_~r   _ItreXWAIS__#       (coded as above)

      Source |       SS       df       MS              Number of obs =     119
-------------+------------------------------           F(  7,   111) =    9.31
       Model |  6282.76755     7  897.538221           Prob > F      =  0.0000
    Residual |  10705.0812   111  96.4421729           R-squared     =  0.3698
-------------+------------------------------           Adj R-squared =  0.3301
       Total |  16987.8487   118   143.96482           Root MSE      =  9.8205

------------------------------------------------------------------------------
   WorryMainMeasure_8WEEK |      Coef.   Std. Err.      t    P>|t|     [95% Conf. Interval]
--------------------------+---------------------------------------------------------------
WorryMainMeasure_Baseline |   .7433942   .1628841     4.56   0.000     .4206284     1.06616
               _ICentre_1 |  -4.035702   2.905079    -1.39   0.168     -9.79231    1.720907
            _IWorryscor_1 |  -7.541349   3.453019    -2.18   0.031    -14.38374   -.6989618
            _ICenXWor_1_1 |   9.122654   3.740799     2.44   0.016     1.710011     16.5353
                _Itreat_1 |  -3.229379   5.026677    -0.64   0.522    -13.19008    6.731318
            WAIS_WMLetter |  -.1250086   .3757401    -0.33   0.740    -.8695627    .6195455
            _ItreXWAIS__1 |  -.4989435   .5608095    -0.89   0.376    -1.610225     .612338
                    _cons |   18.05084   10.31926     1.75   0.083    -2.397465    38.49913
------------------------------------------------------------------------------

. xi: regress WorryMainMeasure_8WEEK WorryMainMeasure_Baseline i.Centre*i.Worryscorestrata
i.treat*Beads_60
i.Centre          _ICentre_0-1        (naturally coded; _ICentre_0 omitted)
i.Worryscores~a   _IWorryscor_0-1     (naturally coded; _IWorryscor_0 omitted)
i.Cen~e*i.Wor~a   _ICenXWor_#_#       (coded as above)
i.treat           _Itreat_0-1         (naturally coded; _Itreat_0 omitted)
i.treat*Bead~60   _ItreXBeads_#       (coded as above)

      Source |       SS       df       MS              Number of obs =     117
-------------+------------------------------           F(  7,   109) =    9.81
       Model |  6427.66055     7  918.237221           Prob > F      =  0.0000
    Residual |  10197.4847   109   93.554906           R-squared     =  0.3866
-------------+------------------------------           Adj R-squared =  0.3472
       Total |  16625.1453   116  143.320218           Root MSE      =  9.6724

------------------------------------------------------------------------------
   WorryMainMeasure_8WEEK |      Coef.   Std. Err.      t    P>|t|     [95% Conf. Interval]
--------------------------+---------------------------------------------------------------
WorryMainMeasure_Baseline |   .7484991   .1635685     4.58   0.000     .4243117    1.072687
               _ICentre_1 |  -3.338045   2.890597    -1.15   0.251    -9.067115    2.391025
            _IWorryscor_1 |  -6.949771   3.498286    -1.99   0.049    -13.88326   -.0162819
            _ICenXWor_1_1 |   8.826724   3.720374     2.37   0.019     1.453065    16.20038
                _Itreat_1 |  -5.490553   2.954342    -1.86   0.066    -11.34596    .3648557
                 Beads_60 |   -.146222   .2067636    -0.71   0.481    -.5560208    .2635767
            _ItreXBeads_1 |  -.2985223   .3135996    -0.95   0.343    -.9200666    .3230219
                    _cons |   17.21508   9.184493     1.87   0.064    -.9882917    35.41844
------------------------------------------------------------------------------

. xi: regress WorryMainMeasure_8WEEK WorryMainMeasure_Baseline i.Centre*i.Worryscorestrata
i.treat*IPQ_CureControl
i.Centre          _ICentre_0-1        (naturally coded; _ICentre_0 omitted)
```

```
i.Worryscores~a   _IWorryscor_0-1     (naturally coded; _IWorryscor_0 omitted)
i.Cen~e*i.Wor~a   _ICenXWor_#_#       (coded as above)
i.treat           _Itreat_0-1         (naturally coded; _Itreat_0 omitted)
i.treat*IPQ_C~l   _ItreXIPQ_C_#       (coded as above)

      Source |       SS       df       MS              Number of obs =     138
-------------+------------------------------           F(  7,   130) =   10.82
       Model |  7130.70237     7  1018.67177           Prob > F      =  0.0000
    Residual |  12244.4643   130  94.1881869           R-squared     =  0.3680
-------------+------------------------------           Adj R-squared =  0.3340
       Total |  19375.1667   137  141.424574           Root MSE      =  9.7051

------------------------------------------------------------------------------------
   WorryMainMeasure_8WEEK |      Coef.   Std. Err.      t    P>|t|     [95% Conf. Interval]
-------------------------+----------------------------------------------------------
WorryMainMeasure_Baseline |   .7195155   .1527749     4.71   0.000     .4172686    1.021762
               _ICentre_1 |  -5.117838   2.617487    -1.96   0.053    -10.29622    .0605481
            _IWorryscor_1 |  -6.620646   3.255519    -2.03   0.044     -13.0613   -.1799901
            _ICenXWor_1_1 |    10.2633   3.385116     3.03   0.003     3.566251    16.96034
                _Itreat_1 |  -8.982053   6.436687    -1.40   0.165    -21.71627    3.752162
          IPQ_CureControl |  -.0320849   .1796816    -0.18   0.859    -.3875634    .3233935
            _ItreXIPQ_C_1 |    .114813   .2687476     0.43   0.670     -.416872    .6464979
                    _cons |   18.32714   9.135661     2.01   0.047      .253331    36.40095
------------------------------------------------------------------------------------

. xi: regress WorryMainMeasure_8WEEK WorryMainMeasure_Baseline i.Centre*i.Worryscorestrata
i.treat*IPQ_Timeline
i.Centre          _ICentre_0-1        (naturally coded; _ICentre_0 omitted)
i.Worryscores~a   _IWorryscor_0-1     (naturally coded; _IWorryscor_0 omitted)
i.Cen~e*i.Wor~a   _ICenXWor_#_#       (coded as above)
i.treat           _Itreat_0-1         (naturally coded; _Itreat_0 omitted)
i.treat*IPQ_T~e   _ItreXIPQ_T_#       (coded as above)

      Source |       SS       df       MS              Number of obs =     138
-------------+------------------------------           F(  7,   130) =   11.20
       Model |  7286.91541     7  1040.98792           Prob > F      =  0.0000
    Residual |  12088.2513   130  92.9865481           R-squared     =  0.3761
-------------+------------------------------           Adj R-squared =  0.3425
       Total |  19375.1667   137  141.424574           Root MSE      =   9.643

------------------------------------------------------------------------------------
   WorryMainMeasure_8WEEK |      Coef.   Std. Err.      t    P>|t|     [95% Conf. Interval]
-------------------------+----------------------------------------------------------
WorryMainMeasure_Baseline |   .6991287   .1508308     4.64   0.000      .400728    .9975294
               _ICentre_1 |  -5.097053   2.571439    -1.98   0.050    -10.18434   -.0097689
            _IWorryscor_1 |  -6.549974   3.231566    -2.03   0.045    -12.94324   -.1567067
            _ICenXWor_1_1 |   9.820496   3.379697     2.91   0.004      3.13417    16.50682
                _Itreat_1 |  -4.762367   5.057202    -0.94   0.348    -14.76744    5.242702
             IPQ_Timeline |  -.3494616   .4696164    -0.74   0.458    -1.278542    .5796183
            _ItreXIPQ_T_1 |  -.2747424   .6916976    -0.40   0.692    -1.643183    1.093699
                    _cons |   21.53404    9.59767     2.24   0.027     2.546194    40.52188
------------------------------------------------------------------------------------

. xi: regress WorryMainMeasure_8WEEK WorryMainMeasure_Baseline i.Centre*i.Worryscorestrata
i.treat*IPQ_StateOfMind
i.Centre          _ICentre_0-1        (naturally coded; _ICentre_0 omitted)
i.Worryscores~a   _IWorryscor_0-1     (naturally coded; _IWorryscor_0 omitted)
i.Cen~e*i.Wor~a   _ICenXWor_#_#       (coded as above)
i.treat           _Itreat_0-1         (naturally coded; _Itreat_0 omitted)
i.treat*IPQ_S~d   _ItreXIPQ_S_#       (coded as above)

      Source |       SS       df       MS              Number of obs =     138
-------------+------------------------------           F(  7,   130) =   10.88
       Model |  7159.13857     7  1022.73408           Prob > F      =  0.0000
    Residual |  12216.0281   130  93.9694469           R-squared     =  0.3695
-------------+------------------------------           Adj R-squared =  0.3356
       Total |  19375.1667   137  141.424574           Root MSE      =  9.6938

------------------------------------------------------------------------------------
   WorryMainMeasure_8WEEK |      Coef.   Std. Err.      t    P>|t|     [95% Conf. Interval]
-------------------------+----------------------------------------------------------
WorryMainMeasure_Baseline |   .7239716    .150976     4.80   0.000     .4252836     1.02266
               _ICentre_1 |  -5.324464   2.584219    -2.06   0.041    -10.43703   -.2118962
            _IWorryscor_1 |  -6.547808   3.262877    -2.01   0.047    -13.00302   -.0925954
            _ICenXWor_1_1 |    10.4515   3.416232     3.06   0.003     3.692893     17.2101
                _Itreat_1 |  -7.369983   4.084755    -1.80   0.074    -15.45118    .7112164
          IPQ_StateOfMind |   .3153761   .9576468     0.33   0.742    -1.579214    2.209966
```

```
          _ItreXIPQ_S_1 |   .4555964    1.57891     0.29   0.773    -2.668089    3.579281
                  _cons |   16.53642   8.906208     1.86   0.066    -1.083449    34.15629
------------------------------------------------------------------------------------------

. xi: regress WorryMainMeasure_8WEEK WorryMainMeasure_Baseline i.Centre*i.Worryscorestrata
i.treat*IPQ_Personality
i.Centre          _ICentre_0-1        (naturally coded; _ICentre_0 omitted)
i.Worryscores~a   _IWorryscor_0-1     (naturally coded; _IWorryscor_0 omitted)
i.Cen~e*i.Wor~a   _ICenXWor_#_#       (coded as above)
i.treat           _Itreat_0-1         (naturally coded; _Itreat_0 omitted)
i.treat*IPQ_P~y   _ItreXIPQ_P_#       (coded as above)

      Source |       SS       df       MS              Number of obs =     138
-------------+------------------------------           F(  7,   130) =   11.11
       Model |  7250.35978     7  1035.76568           Prob > F      =  0.0000
    Residual |  12124.8069   130  93.2677453           R-squared     =  0.3742
-------------+------------------------------           Adj R-squared =  0.3405
       Total |  19375.1667   137  141.424574           Root MSE      =  9.6575

------------------------------------------------------------------------------------------
   WorryMainMeasure_8WEEK |      Coef.   Std. Err.      t    P>|t|     [95% Conf. Interval]
--------------------------+---------------------------------------------------------------
WorryMainMeasure_Baseline |   .7160616   .1503656     4.76   0.000     .4185813    1.013542
               _ICentre_1 |  -4.956731    2.59259    -1.91   0.058    -10.08586    .1723979
            _IWorryscor_1 |  -6.195385   3.257331    -1.90   0.059    -12.63962    .2488551
            _ICenXWor_1_1 |   10.08057   3.360864     3.00   0.003     3.431502    16.72964
                _Itreat_1 |   -10.5947   3.940967    -2.69   0.008    -18.39143   -2.797965
          IPQ_Personality |  -.6492905   .9487172    -0.68   0.495    -2.526214    1.227633
            _ItreXIPQ_P_1 |   1.599144   1.338724     1.19   0.234    -1.049362     4.24765
                    _cons |   19.29075   9.020608     2.14   0.034     1.444555    37.13694
------------------------------------------------------------------------------------------

. xi: regress WorryMainMeasure_8WEEK WorryMainMeasure_Baseline i.Centre*i.Worryscorestrata
i.treat*MAP_AlcoholDays
i.Centre          _ICentre_0-1        (naturally coded; _ICentre_0 omitted)
i.Worryscores~a   _IWorryscor_0-1     (naturally coded; _IWorryscor_0 omitted)
i.Cen~e*i.Wor~a   _ICenXWor_#_#       (coded as above)
i.treat           _Itreat_0-1         (naturally coded; _Itreat_0 omitted)
i.treat*MAP_A~s   _ItreXMAP_A_#       (coded as above)

      Source |       SS       df       MS              Number of obs =     143
-------------+------------------------------           F(  7,   135) =   11.29
       Model |  7279.03735     7  1039.86248           Prob > F      =  0.0000
    Residual |  12434.7109   135  92.1089696           R-squared     =  0.3692
-------------+------------------------------           Adj R-squared =  0.3365
       Total |  19713.7483   142  138.829213           Root MSE      =  9.5973

------------------------------------------------------------------------------------------
   WorryMainMeasure_8WEEK |      Coef.   Std. Err.      t    P>|t|     [95% Conf. Interval]
--------------------------+---------------------------------------------------------------
WorryMainMeasure_Baseline |   .7327269   .1516608     4.83   0.000     .4327884    1.032665
               _ICentre_1 |  -5.040735   2.487915    -2.03   0.045    -9.961065    -.120404
            _IWorryscor_1 |  -6.652192   3.237715    -2.05   0.042     -13.0554   -.2489873
            _ICenXWor_1_1 |   10.05986   3.289717     3.06   0.003     3.553807     16.5659
                _Itreat_1 |  -5.069484   1.920556    -2.64   0.009    -8.867752   -1.271215
          MAP_AlcoholDays |   .1452168    .142439     1.02   0.310    -.1364836    .4269173
            _ItreXMAP_A_1 |  -.2411039   .1980082    -1.22   0.225    -.6327032    .1504953
                    _cons |   16.20357   8.817152     1.84   0.068    -1.234048    33.64118
------------------------------------------------------------------------------------------

. xi: regress WorryMainMeasure_8WEEK WorryMainMeasure_Baseline i.Centre*i.Worryscorestrata
i.treat*MAP_AlcoholAmount
i.Centre          _ICentre_0-1        (naturally coded; _ICentre_0 omitted)
i.Worryscores~a   _IWorryscor_0-1     (naturally coded; _IWorryscor_0 omitted)
i.Cen~e*i.Wor~a   _ICenXWor_#_#       (coded as above)
i.treat           _Itreat_0-1         (naturally coded; _Itreat_0 omitted)
i.treat*MAP_A~t   _ItreXMAP_A_#       (coded as above)

      Source |       SS       df       MS              Number of obs =     143
-------------+------------------------------           F(  7,   135) =   11.85
       Model |  7502.63233     7  1071.80462           Prob > F      =  0.0000
    Residual |  12211.1159   135  90.4527105           R-squared     =  0.3806
-------------+------------------------------           Adj R-squared =  0.3485
       Total |  19713.7483   142  138.829213           Root MSE      =  9.5107

------------------------------------------------------------------------------------------
   WorryMainMeasure_8WEEK |      Coef.   Std. Err.      t    P>|t|     [95% Conf. Interval]
```

```
-------------------------+----------------------------------------------------------------
WorryMainMeasure_Baseline |   .7273762   .1472004     4.94   0.000     .4362591    1.018493
               _ICentre_1 |  -5.503111   2.478762    -2.22   0.028    -10.40534   -.6008817
           _IWorryscor_1 |  -6.727281   3.154036    -2.13   0.035    -12.96499   -.4895689
           _ICenXWor_1_1 |   10.21867   3.237055     3.16   0.002     3.816772    16.62057
               _Itreat_1 |   -8.39172    1.89352    -4.43   0.000    -12.13652    -4.64692
       MAP_AlcoholAmount |  -.1519055   .1551586    -0.98   0.329    -.4587613    .1549504
           _ItreXMAP_A_1 |   .4648569   .2364311     1.97   0.051     -.002731    .9324448
                   _cons |   18.13268   8.423811     2.15   0.033     1.472969    34.79238
------------------------------------------------------------------------------------------

. xi: regress WorryMainMeasure_8WEEK WorryMainMeasure_Baseline i.Centre*i.Worryscorestrata
i.treat*MAP_AlcoholPastYear
i.Centre          _ICentre_0-1        (naturally coded; _ICentre_0 omitted)
i.Worryscores~a   _IWorryscor_0-1     (naturally coded; _IWorryscor_0 omitted)
i.Cen~e*i.Wor~a   _ICenXWor_#_#       (coded as above)
i.treat           _Itreat_0-1         (naturally coded; _Itreat_0 omitted)
i.treat*MAP_A~r   _ItreXMAP_A_#       (coded as above)

      Source |       SS       df       MS              Number of obs =     143
-------------+------------------------------           F(  7,   135) =   11.07
       Model |  7189.17008     7  1027.0243            Prob > F      =  0.0000
    Residual |  12524.5782   135  92.7746531           R-squared     =  0.3647
-------------+------------------------------           Adj R-squared =  0.3317
       Total |  19713.7483   142  138.829213           Root MSE      =   9.632

------------------------------------------------------------------------------------------
   WorryMainMeasure_8WEEK |      Coef.   Std. Err.      t    P>|t|     [95% Conf. Interval]
-------------------------+----------------------------------------------------------------
WorryMainMeasure_Baseline |   .7331931   .1505972     4.87   0.000     .4353582    1.031028
               _ICentre_1 |  -5.332493   2.544259    -2.10   0.038    -10.36425   -.3007314
           _IWorryscor_1 |  -6.699209   3.251047    -2.06   0.041    -13.12878   -.2696379
           _ICenXWor_1_1 |   10.28744   3.343267     3.08   0.003     3.675491     16.8994
               _Itreat_1 |  -7.596878   2.918415    -2.60   0.010     -13.3686   -1.825152
     MAP_AlcoholPastYear |   .1485492   2.451442     0.06   0.952    -4.699649    4.996747
           _ItreXMAP_A_1 |   1.774483   3.504981     0.51   0.613    -5.157291    8.706257
                   _cons |   16.94448   8.773248     1.93   0.056    -.4063068    34.29526
------------------------------------------------------------------------------------------

. xi: regress WorryMainMeasure_8WEEK WorryMainMeasure_Baseline i.Centre*i.Worryscorestrata
i.treat*Cannabis_pastmonth
i.Centre          _ICentre_0-1        (naturally coded; _ICentre_0 omitted)
i.Worryscores~a   _IWorryscor_0-1     (naturally coded; _IWorryscor_0 omitted)
i.Cen~e*i.Wor~a   _ICenXWor_#_#       (coded as above)
i.treat           _Itreat_0-1         (naturally coded; _Itreat_0 omitted)
i.treat*Canna~h   _ItreXCanna_#       (coded as above)

      Source |       SS       df       MS              Number of obs =     143
-------------+------------------------------           F(  7,   135) =   11.36
       Model |   7308.4868     7  1044.06954           Prob > F      =  0.0000
    Residual |  12405.2615   135  91.8908256           R-squared     =  0.3707
-------------+------------------------------           Adj R-squared =  0.3381
       Total |  19713.7483   142  138.829213           Root MSE      =   9.586

------------------------------------------------------------------------------------------
   WorryMainMeasure_8WEEK |      Coef.   Std. Err.      t    P>|t|     [95% Conf. Interval]
-------------------------+----------------------------------------------------------------
WorryMainMeasure_Baseline |   .7492769   .1512166     4.95   0.000     .4502169    1.048337
               _ICentre_1 |  -5.348775   2.497167    -2.14   0.034     -10.2874   -.4101476
           _IWorryscor_1 |  -7.180646   3.273906    -2.19   0.030    -13.65542   -.7058673
           _ICenXWor_1_1 |   10.59209   3.302054     3.21   0.002     4.061645    17.12254
               _Itreat_1 |  -6.172686   1.678744    -3.68   0.000    -9.492726   -2.852647
      Cannabis_pastmonth |   4.473347    3.84382     1.16   0.247    -3.128546    12.07524
           _ItreXCanna_1 |  -1.065392   5.903219    -0.18   0.857    -12.74014    10.60936
                   _cons |   15.77604    8.62988     1.83   0.070    -1.291208    32.84328
------------------------------------------------------------------------------------------

. xi: regress WorryMainMeasure_8WEEK WorryMainMeasure_Baseline i.Centre*i.Worryscorestrata
i.treat*CannabisPastYear
i.Centre          _ICentre_0-1        (naturally coded; _ICentre_0 omitted)
i.Worryscores~a   _IWorryscor_0-1     (naturally coded; _IWorryscor_0 omitted)
i.Cen~e*i.Wor~a   _ICenXWor_#_#       (coded as above)
i.treat           _Itreat_0-1         (naturally coded; _Itreat_0 omitted)
i.treat*Canna~r   _ItreXCanna_#       (coded as above)

      Source |       SS       df       MS              Number of obs =     143
-------------+------------------------------           F(  7,   135) =   11.10
```

```
       Model |  7201.25242     7  1028.75035           Prob > F      =  0.0000
    Residual |  12512.4958   135  92.6851543           R-squared     =  0.3653
-------------+------------------------------           Adj R-squared =  0.3324
       Total |  19713.7483   142  138.829213           Root MSE      =  9.6273

------------------------------------------------------------------------------------------
   WorryMainMeasure_8WEEK |      Coef.   Std. Err.      t    P>|t|     [95% Conf. Interval]
--------------------------+---------------------------------------------------------------
WorryMainMeasure_Baseline |   .7320765   .1501001     4.88   0.000     .4352248    1.028928
               _ICentre_1 |  -5.272067   2.514703    -2.10   0.038    -10.24538   -.2987594
            _IWorryscor_1 |  -6.709383   3.231571    -2.08   0.040    -13.10044   -.3183309
            _ICenXWor_1_1 |    10.2785   3.306332     3.11   0.002     3.739592    16.81741
                _Itreat_1 |  -6.348954   1.725591    -3.68   0.000    -9.761642   -2.936266
         CannabisPastYear |   2.110701   3.457106     0.61   0.543    -4.726391    8.947792
            _ItreXCanna_1 |  -.1123098   4.867421    -0.02   0.982    -9.738571    9.513952
                    _cons |   16.83717   8.587068     1.96   0.052    -.1454125    33.81974
------------------------------------------------------------------------------------------

. xi: regress WorryMainMeasure_8WEEK WorryMainMeasure_Baseline i.Centre*i.Worryscorestrata
i.treat*IlllicitDrugs_pastmonth
i.Centre          _ICentre_0-1        (naturally coded; _ICentre_0 omitted)
i.Worryscores~a   _IWorryscor_0-1     (naturally coded; _IWorryscor_0 omitted)
i.Cen~e*i.Wor~a   _ICenXWor_#_#       (coded as above)
i.treat           _Itreat_0-1         (naturally coded; _Itreat_0 omitted)
i.treat*Illli~h   _ItreXIllli_#       (coded as above)

      Source |       SS       df       MS              Number of obs =     143
-------------+------------------------------           F(  7,   135) =   11.17
       Model |   7229.5317     7  1032.79024           Prob > F      =  0.0000
    Residual |  12484.2165   135  92.4756781           R-squared     =  0.3667
-------------+------------------------------           Adj R-squared =  0.3339
       Total |  19713.7483   142  138.829213           Root MSE      =  9.6164

------------------------------------------------------------------------------------------
   WorryMainMeasure_8WEEK |      Coef.   Std. Err.      t    P>|t|     [95% Conf. Interval]
--------------------------+---------------------------------------------------------------
WorryMainMeasure_Baseline |   .7359611   .1511236     4.87   0.000     .4370852    1.034837
               _ICentre_1 |   -5.16578    2.49827    -2.07   0.041    -10.10659   -.2249709
            _IWorryscor_1 |  -6.715757   3.272242    -2.05   0.042    -13.18725   -.2442684
            _ICenXWor_1_1 |   10.15477   3.278884     3.10   0.002     3.670151     16.6394
                _Itreat_1 |  -6.813654   1.678253    -4.06   0.000    -10.13272   -3.494586
  IlllicitDrugs_pastmonth |  -2.134091   4.526605    -0.47   0.638    -11.08632    6.818141
            _ItreXIllli_1 |   5.791932   6.102017     0.95   0.344     -6.27598    17.85984
                    _cons |   16.98075   8.608569     1.97   0.051    -.0443495    34.00585
------------------------------------------------------------------------------------------

. xi: regress WorryMainMeasure_8WEEK WorryMainMeasure_Baseline i.Centre*i.Worryscorestrata
i.treat*IllicitDrugs_PastYear
i.Centre          _ICentre_0-1        (naturally coded; _ICentre_0 omitted)
i.Worryscores~a   _IWorryscor_0-1     (naturally coded; _IWorryscor_0 omitted)
i.Cen~e*i.Wor~a   _ICenXWor_#_#       (coded as above)
i.treat           _Itreat_0-1         (naturally coded; _Itreat_0 omitted)
i.treat*Illic~r   _ItreXIllic_#       (coded as above)

      Source |       SS       df       MS              Number of obs =     143
-------------+------------------------------           F(  7,   135) =   11.57
       Model |  7390.77592     7  1055.82513           Prob > F      =  0.0000
    Residual |  12322.9723   135  91.2812765           R-squared     =  0.3749
-------------+------------------------------           Adj R-squared =  0.3425
       Total |  19713.7483   142  138.829213           Root MSE      =  9.5541

------------------------------------------------------------------------------------------
   WorryMainMeasure_8WEEK |      Coef.   Std. Err.      t    P>|t|     [95% Conf. Interval]
--------------------------+---------------------------------------------------------------
WorryMainMeasure_Baseline |   .7209355   .1480274     4.87   0.000     .4281829    1.013688
               _ICentre_1 |  -5.206565   2.478714    -2.10   0.038     -10.1087    -.304431
            _IWorryscor_1 |   -6.03453   3.180208    -1.90   0.060      -12.324    .2549434
            _ICenXWor_1_1 |   9.816471   3.247798     3.02   0.003     3.393327    16.23962
                _Itreat_1 |  -7.230604   1.714159    -4.22   0.000    -10.62068   -3.840525
    IllicitDrugs_PastYear |  -5.661141   3.626463    -1.56   0.121    -12.83317    1.510887
            _ItreXIllic_1 |   7.511976   4.868654     1.54   0.125    -2.116724    17.14068
                    _cons |   18.14832   8.457564     2.15   0.034     1.421864    34.87478
------------------------------------------------------------------------------------------

. xi: regress WorryMainMeasure_8WEEK WorryMainMeasure_Baseline i.Centre*i.Worryscorestrata
i.treat*age
i.Centre          _ICentre_0-1        (naturally coded; _ICentre_0 omitted)
```

```
i.Worryscores~a   _IWorryscor_0-1     (naturally coded; _IWorryscor_0 omitted)
i.Cen~e*i.Wor~a   _ICenXWor_#_#       (coded as above)
i.treat           _Itreat_0-1         (naturally coded; _Itreat_0 omitted)
i.treat*age       _ItreXage_#         (coded as above)

      Source |       SS       df       MS              Number of obs =     143
-------------+------------------------------           F(  7,   135) =   11.05
       Model |  7180.78426     7  1025.82632           Prob > F      =  0.0000
    Residual |   12532.964   135  92.8367703           R-squared     =  0.3643
-------------+------------------------------           Adj R-squared =  0.3313
       Total |  19713.7483   142  138.829213           Root MSE      =  9.6352

------------------------------------------------------------------------------------------
   WorryMainMeasure_8WEEK |      Coef.   Std. Err.      t    P>|t|     [95% Conf. Interval]
--------------------------+---------------------------------------------------------------
WorryMainMeasure_Baseline |   .7244206   .1497716     4.84   0.000     .4282184    1.020623
               _ICentre_1 |  -4.997177     2.5011    -2.00   0.048    -9.943584     -.05077
            _IWorryscor_1 |  -6.341879   3.233349    -1.96   0.052    -12.73645    .0526905
            _ICenXWor_1_1 |   9.888898   3.272711     3.02   0.003     3.416483    16.36131
                _Itreat_1 |  -2.261062    6.26908    -0.36   0.719    -14.65937    10.13725
                      age |   .0328393   .0950625     0.35   0.730    -.1551651    .2208436
              _ItreXage_1 |  -.0985558   .1455774    -0.68   0.500    -.3864631    .1893514
                    _cons |   15.96106   9.487002     1.68   0.095    -2.801315    34.72343
------------------------------------------------------------------------------------------

. xi: regress WorryMainMeasure_8WEEK WorryMainMeasure_Baseline i.Centre*i.Worryscorestrata
i.treat*sex
i.Centre          _ICentre_0-1        (naturally coded; _ICentre_0 omitted)
i.Worryscores~a   _IWorryscor_0-1     (naturally coded; _IWorryscor_0 omitted)
i.Cen~e*i.Wor~a   _ICenXWor_#_#       (coded as above)
i.treat           _Itreat_0-1         (naturally coded; _Itreat_0 omitted)
i.treat*sex       _ItreXsex_#         (coded as above)

      Source |       SS       df       MS              Number of obs =     143
-------------+------------------------------           F(  7,   135) =   11.53
       Model |  7375.89899     7  1053.69986           Prob > F      =  0.0000
    Residual |  12337.8493   135   91.391476           R-squared     =  0.3742
-------------+------------------------------           Adj R-squared =  0.3417
       Total |  19713.7483   142  138.829213           Root MSE      =  9.5599

------------------------------------------------------------------------------------------
   WorryMainMeasure_8WEEK |      Coef.   Std. Err.      t    P>|t|     [95% Conf. Interval]
--------------------------+---------------------------------------------------------------
WorryMainMeasure_Baseline |   .7033765   .1486572     4.73   0.000     .4093783    .9973747
               _ICentre_1 |  -5.212224    2.48262    -2.10   0.038    -10.12208   -.3023663
            _IWorryscor_1 |  -5.787171   3.175508    -1.82   0.071    -12.06735    .4930056
            _ICenXWor_1_1 |   9.351654   3.271384     2.86   0.005     2.881863    15.82145
                _Itreat_1 |   .1855793   4.939858     0.04   0.970     -9.58394    9.955099
                      sex |    .899063   2.278373     0.39   0.694    -3.606857    5.404983
              _ItreXsex_1 |  -4.650087   3.307989    -1.41   0.162    -11.19227    1.892097
                    _cons |   17.38076   8.785147     1.98   0.050      .006442    34.75508
------------------------------------------------------------------------------------------

. xi: regress WorryMainMeasure_8WEEK WorryMainMeasure_Baseline i.Centre*i.Worryscorestrata
i.treat*i.ethnicity
i.Centre          _ICentre_0-1        (naturally coded; _ICentre_0 omitted)
i.Worryscores~a   _IWorryscor_0-1     (naturally coded; _IWorryscor_0 omitted)
i.Cen~e*i.Wor~a   _ICenXWor_#_#       (coded as above)
i.treat           _Itreat_0-1         (naturally coded; _Itreat_0 omitted)
i.ethnicity       _Iethnicity_1-7     (naturally coded; _Iethnicity_1 omitted)
i.treat*i.eth~y   _ItreXeth_#_#       (coded as above)
note: _ItreXeth_1_4 omitted because of collinearity
note: _ItreXeth_1_5 omitted because of collinearity

      Source |       SS       df       MS              Number of obs =     143
-------------+------------------------------           F(  9,   133) =    8.53
       Model |  7216.98604     9  801.887337           Prob > F      =  0.0000
    Residual |  12496.7622   133  93.9606182           R-squared     =  0.3661
-------------+------------------------------           Adj R-squared =  0.3232
       Total |  19713.7483   142  138.829213           Root MSE      =  9.6933

------------------------------------------------------------------------------------------
   WorryMainMeasure_8WEEK |      Coef.   Std. Err.      t    P>|t|     [95% Conf. Interval]
--------------------------+---------------------------------------------------------------
WorryMainMeasure_Baseline |   .6900266   .1581292     4.36   0.000     .3772531      1.0028
               _ICentre_1 |  -4.820849   2.578237    -1.87   0.064    -9.920502    .2788039
            _IWorryscor_1 |  -5.938396   3.302857    -1.80   0.074    -12.47132    .5945271
```

```
           _ICenXWor_1_1 |   9.750917   3.319708     2.94   0.004     3.184664    16.31717
               _Itreat_1 |  -6.545156    1.70293    -3.84   0.000    -9.913485   -3.176827
         _Iethnicity_4 |   4.966547   9.861888     0.50   0.615    -14.53988    24.47298
         _Iethnicity_5 |  -3.959816   6.045856    -0.65   0.514    -15.91828    7.998653
         _Iethnicity_7 |  -.7413881   4.233942    -0.18   0.861    -9.115961    7.633184
         _ItreXeth_1_4 |          0  (omitted)
         _ItreXeth_1_5 |          0  (omitted)
         _ItreXeth_1_7 |   -1.31466   7.155057    -0.18   0.854    -15.46709    12.83777
                 _cons |   19.52512   8.992451     2.17   0.032     1.738397    37.31184
------------------------------------------------------------------------------------------

. xi: regress WorryMainMeasure_8WEEK WorryMainMeasure_Baseline i.Centre*i.Worryscorestrata
i.treat*i.Diagnosis
i.Centre          _ICentre_0-1        (naturally coded; _ICentre_0 omitted)
i.Worryscores~a   _IWorryscor_0-1     (naturally coded; _IWorryscor_0 omitted)
i.Cen~e*i.Wor~a   _ICenXWor_#_#       (coded as above)
i.treat           _Itreat_0-1         (naturally coded; _Itreat_0 omitted)
i.Diagnosis       _IDiagnosis_1-4     (naturally coded; _IDiagnosis_1 omitted)
i.treat*i.Dia~s   _ItreXDia_#_#       (coded as above)

      Source |       SS       df       MS              Number of obs =     143
-------------+------------------------------           F( 11,   131) =    7.26
       Model |  7468.24691    11  678.931537           Prob > F      =  0.0000
    Residual |  12245.5013   131  93.4771095           R-squared     =  0.3788
-------------+------------------------------           Adj R-squared =  0.3267
       Total |  19713.7483   142  138.829213           Root MSE      =  9.6684

------------------------------------------------------------------------------------------
   WorryMainMeasure_8WEEK |      Coef.   Std. Err.      t    P>|t|     [95% Conf. Interval]
--------------------------+---------------------------------------------------------------
WorryMainMeasure_Baseline |   .6829027   .1548727     4.41   0.000     .3765276    .9892779
               _ICentre_1 |  -5.307296   2.556888    -2.08   0.040    -10.36543   -.2491626
            _IWorryscor_1 |  -6.064382   3.241321    -1.87   0.064    -12.47649    .3477247
            _ICenXWor_1_1 |   9.633688   3.325954     2.90   0.004     3.054157    16.21322
                _Itreat_1 |   -6.02356    1.88947    -3.19   0.002    -9.761381   -2.285738
            _IDiagnosis_2 |    5.85658   4.337733     1.35   0.179    -2.724489    14.43765
            _IDiagnosis_3 |  -1.553311   4.635186    -0.34   0.738    -10.72281    7.616192
            _IDiagnosis_4 |  -.1919556   3.271166    -0.06   0.953    -6.663103    6.279192
            _ItreXDia_1_2 |    -2.8297   6.285178    -0.45   0.653    -15.26328    9.603882
            _ItreXDia_1_3 |  -4.006323   7.330631    -0.55   0.586    -18.50806    10.49541
            _ItreXDia_1_4 |   .7306963   5.336455     0.14   0.891    -9.826085    11.28748
                    _cons |   19.76593   8.741849     2.26   0.025     2.472471     37.0594
------------------------------------------------------------------------------------------

. xi: regress WorryMainMeasure_8WEEK WorryMainMeasure_Baseline i.Centre*i.Worryscorestrata
i.treat*i.Employment
i.Centre          _ICentre_0-1        (naturally coded; _ICentre_0 omitted)
i.Worryscores~a   _IWorryscor_0-1     (naturally coded; _IWorryscor_0 omitted)
i.Cen~e*i.Wor~a   _ICenXWor_#_#       (coded as above)
i.treat           _Itreat_0-1         (naturally coded; _Itreat_0 omitted)
i.Employment      _IEmploymen_1-7     (naturally coded; _IEmploymen_1 omitted)
i.treat*i.Emp~t   _ItreXEmp_#_#       (coded as above)
note: _ItreXEmp_1_6 omitted because of collinearity

      Source |       SS       df       MS              Number of obs =     143
-------------+------------------------------           F( 16,   126) =    5.78
       Model |  8343.64941    16  521.478088           Prob > F      =  0.0000
    Residual |  11370.0988   126  90.2388797           R-squared     =  0.4232
-------------+------------------------------           Adj R-squared =  0.3500
       Total |  19713.7483   142  138.829213           Root MSE      =  9.4994

------------------------------------------------------------------------------------------
   WorryMainMeasure_8WEEK |      Coef.   Std. Err.      t    P>|t|     [95% Conf. Interval]
--------------------------+---------------------------------------------------------------
WorryMainMeasure_Baseline |   .7081324   .1524823     4.64   0.000     .4063744     1.00989
               _ICentre_1 |  -4.851919   2.526512    -1.92   0.057    -9.851812    .1479732
            _IWorryscor_1 |   -5.77886   3.390416    -1.70   0.091    -12.48839    .9306732
            _ICenXWor_1_1 |   8.244526   3.376393     2.44   0.016     1.562743    14.92631
                _Itreat_1 |  -7.881353    6.32116    -1.25   0.215    -20.39074    4.628038
           _IEmploymen_2 |   2.477022   5.123168     0.48   0.630    -7.661577    12.61562
           _IEmploymen_3 |   2.659322   3.380241     0.79   0.433    -4.030076     9.34872
           _IEmploymen_4 |  -3.389954   4.922088    -0.69   0.492    -13.13062    6.350713
           _IEmploymen_5 |  -3.424215   10.12133    -0.34   0.736    -23.45403     16.6056
           _IEmploymen_6 |  -3.472681   8.023905    -0.43   0.666    -19.35175    12.40639
           _IEmploymen_7 |  -6.272983   7.408204    -0.85   0.399     -20.9336    8.387636
            _ItreXEmp_1_2 |  -5.795092   8.247389    -0.70   0.484    -22.11643    10.52625
            _ItreXEmp_1_3 |   1.443819   6.621592     0.22   0.828    -11.66012    14.54776
```

```
        _ItreXEmp_1_4 |   11.23982   9.986547     1.13   0.263    -8.523261    31.00291
        _ItreXEmp_1_5 |   .4220149   14.85002     0.03   0.977    -28.96574    29.80977
        _ItreXEmp_1_6 |          0  (omitted)
        _ItreXEmp_1_7 |   25.38059   13.38611     1.90   0.060    -1.110141    51.87131
                _cons |   17.05007   9.373315     1.82   0.071    -1.499447    35.59958
------------------------------------------------------------------------------------

. xi: regress WorryMainMeasure_8WEEK WorryMainMeasure_Baseline i.Centre*i.Worryscorestrata
i.treat*WASI_IQ
i.Centre          _ICentre_0-1        (naturally coded; _ICentre_0 omitted)
i.Worryscores~a   _IWorryscor_0-1     (naturally coded; _IWorryscor_0 omitted)
i.Cen~e*i.Wor~a   _ICenXWor_#_#       (coded as above)
i.treat           _Itreat_0-1         (naturally coded; _Itreat_0 omitted)
i.treat*WASI_IQ   _ItreXWASI__#       (coded as above)

      Source |       SS       df       MS              Number of obs =     133
-------------+------------------------------           F(  7,   125) =   10.88
       Model |  6727.16998     7  961.024283           Prob > F      =  0.0000
    Residual |  11039.7623   125  88.3180988           R-squared     =  0.3786
-------------+------------------------------           Adj R-squared =  0.3438
       Total |  17766.9323   132  134.597972           Root MSE      =  9.3978

------------------------------------------------------------------------------------
   WorryMainMeasure_8WEEK |      Coef.   Std. Err.      t    P>|t|     [95% Conf. Interval]
-------------------------+----------------------------------------------------------
WorryMainMeasure_Baseline |   .7391575   .1490852     4.96   0.000     .4440993    1.034216
               _ICentre_1 |  -3.290818   2.599464    -1.27   0.208     -8.43548    1.853844
            _IWorryscor_1 |  -6.986153   3.196374    -2.19   0.031    -13.31217   -.6601324
            _ICenXWor_1_1 |   9.152653   3.375555     2.71   0.008     2.472011     15.8333
                _Itreat_1 |   10.44797   9.373177     1.11   0.267    -8.102713    28.99865
                  WASI_IQ |   .0304555    .064634     0.47   0.638    -.0974633    .1583743
            _ItreXWASI__1 |  -.1665328   .0911905    -1.83   0.070    -.3470101    .0139444
                    _cons |   12.83623   10.95979     1.17   0.244    -8.854558    34.52702
------------------------------------------------------------------------------------

. xi: regress WorryMainMeasure_8WEEK WorryMainMeasure_Baseline i.Centre*i.Worryscorestrata
i.treat*CPZ_equivalent_Baseline
i.Centre          _ICentre_0-1        (naturally coded; _ICentre_0 omitted)
i.Worryscores~a   _IWorryscor_0-1     (naturally coded; _IWorryscor_0 omitted)
i.Cen~e*i.Wor~a   _ICenXWor_#_#       (coded as above)
i.treat           _Itreat_0-1         (naturally coded; _Itreat_0 omitted)
i.treat*CPZ_e~e   _ItreXCPZ_e_#       (coded as above)

      Source |       SS       df       MS              Number of obs =     142
-------------+------------------------------           F(  7,   134) =   11.48
       Model |   7326.8253     7  1046.68933           Prob > F      =  0.0000
    Residual |  12215.6324   134  91.1614361           R-squared     =  0.3749
-------------+------------------------------           Adj R-squared =  0.3423
       Total |  19542.4577   141  138.598991           Root MSE      =  9.5478

------------------------------------------------------------------------------------
   WorryMainMeasure_8WEEK |      Coef.   Std. Err.      t    P>|t|     [95% Conf. Interval]
-------------------------+----------------------------------------------------------
WorryMainMeasure_Baseline |   .7013734   .1485477     4.72   0.000     .4075719    .9951748
               _ICentre_1 |  -5.507373   2.501936    -2.20   0.029    -10.45577   -.5589808
            _IWorryscor_1 |  -5.620043   3.183086    -1.77   0.080    -11.91563     .675546
            _ICenXWor_1_1 |   10.27319   3.275597     3.14   0.002     3.794635    16.75175
                _Itreat_1 |  -7.076508   2.582715    -2.74   0.007    -12.18467   -1.968347
  CPZ_equivalent_Baseline |   .0017841    .002667     0.67   0.505    -.0034909     .007059
            _ItreXCPZ_e_1 |   .0016025   .0039435     0.41   0.685     -.006197     .009402
                    _cons |   17.49204   8.667033     2.02   0.046     .3501541    34.63392
------------------------------------------------------------------------------------

. xi: regress WorryMainMeasure_8WEEK WorryMainMeasure_Baseline i.Centre*i.Worryscorestrata
i.treat*WorryMainMeasure_Baseline
i.Centre          _ICentre_0-1        (naturally coded; _ICentre_0 omitted)
i.Worryscores~a   _IWorryscor_0-1     (naturally coded; _IWorryscor_0 omitted)
i.Cen~e*i.Wor~a   _ICenXWor_#_#       (coded as above)
i.treat           _Itreat_0-1         (naturally coded; _Itreat_0 omitted)
i.treat*Worry~e   _ItreXWorry_#       (coded as above)
note: WorryMainMeasure_Baseline omitted because of collinearity

      Source |       SS       df       MS              Number of obs =     143
-------------+------------------------------           F(  6,   136) =   13.60
       Model |  7392.77228     6  1232.12871           Prob > F      =  0.0000
    Residual |   12320.976   136  90.5954115           R-squared     =  0.3750
-------------+------------------------------           Adj R-squared =  0.3474
```

```
       Total |  19713.7483   142  138.829213           Root MSE      =  9.5182

------------------------------------------------------------------------------------------
   WorryMainMeasure_8WEEK |      Coef.   Std. Err.      t    P>|t|     [95% Conf. Interval]
--------------------------+---------------------------------------------------------------
WorryMainMeasure_Baseline |    .835008   .1631037     5.12   0.000     .5124604    1.157555
               _ICentre_1 |  -5.045471   2.466796    -2.05   0.043    -9.923709   -.1672329
            _IWorryscor_1 |    -5.8918   3.154601    -1.87   0.064    -12.13022    .3466155
            _ICenXWor_1_1 |   10.00366   3.233707     3.09   0.002     3.608811    16.39851
                _Itreat_1 |   13.09076    11.6801     1.12   0.264    -10.00735    36.18887
WorryMainMeasure_Baseline |          0  (omitted)
            _ItreXWorry_1 |  -.2998518   .1784943    -1.68   0.095    -.6528351    .0531315
                    _cons |   9.924537   9.630865     1.03   0.305    -9.121084    28.97016
------------------------------------------------------------------------------------------

. xi: regress WorryMainMeasure_8WEEK WorryMainMeasure_Baseline i.Centre*i.Worryscorestrata
i.treat*MAP_AlcoholAmount
i.Centre          _ICentre_0-1        (naturally coded; _ICentre_0 omitted)
i.Worryscores~a   _IWorryscor_0-1     (naturally coded; _IWorryscor_0 omitted)
i.Cen~e*i.Wor~a   _ICenXWor_#_#       (coded as above)
i.treat           _Itreat_0-1         (naturally coded; _Itreat_0 omitted)
i.treat*MAP_A~t   _ItreXMAP_A_#       (coded as above)

      Source |       SS       df       MS              Number of obs =     143
-------------+------------------------------           F(  7,   135) =   11.85
       Model |  7502.63233     7  1071.80462           Prob > F      =  0.0000
    Residual |  12211.1159   135  90.4527105           R-squared     =  0.3806
-------------+------------------------------           Adj R-squared =  0.3485
       Total |  19713.7483   142  138.829213           Root MSE      =  9.5107

------------------------------------------------------------------------------------------
   WorryMainMeasure_8WEEK |      Coef.   Std. Err.      t    P>|t|     [95% Conf. Interval]
--------------------------+---------------------------------------------------------------
WorryMainMeasure_Baseline |   .7273762   .1472004     4.94   0.000     .4362591    1.018493
               _ICentre_1 |  -5.503111   2.478762    -2.22   0.028    -10.40534   -.6008817
            _IWorryscor_1 |  -6.727281   3.154036    -2.13   0.035    -12.96499   -.4895689
            _ICenXWor_1_1 |   10.21867   3.237055     3.16   0.002     3.816772    16.62057
                _Itreat_1 |   -8.39172    1.89352    -4.43   0.000    -12.13652    -4.64692
        MAP_AlcoholAmount |  -.1519055   .1551586    -0.98   0.329    -.4587613    .1549504
            _ItreXMAP_A_1 |   .4648569   .2364311     1.97   0.051     -.002731    .9324448
                    _cons |   18.13268   8.423811     2.15   0.033     1.472969    34.79238
------------------------------------------------------------------------------------------

. xi: regress PSYRATS_total_8WEEK PSYRATS_total_Baseline i.Centre*i.Worryscorestrata
i.treat*MAP_AlcoholAmount
i.Centre          _ICentre_0-1        (naturally coded; _ICentre_0 omitted)
i.Worryscores~a   _IWorryscor_0-1     (naturally coded; _IWorryscor_0 omitted)
i.Cen~e*i.Wor~a   _ICenXWor_#_#       (coded as above)
i.treat           _Itreat_0-1         (naturally coded; _Itreat_0 omitted)
i.treat*MAP_A~t   _ItreXMAP_A_#       (coded as above)

      Source |       SS       df       MS              Number of obs =     143
-------------+------------------------------           F(  7,   135) =   11.05
       Model |   1298.8105     7  185.544357           Prob > F      =  0.0000
    Residual |  2267.00768   135  16.7926495           R-squared     =  0.3642
-------------+------------------------------           Adj R-squared =  0.3313
       Total |  3565.81818   142  25.1113956           Root MSE      =  4.0979

---------------------------------------------------------------------------------------
   PSYRATS_total_8WEEK |      Coef.   Std. Err.      t    P>|t|     [95% Conf. Interval]
-----------------------+---------------------------------------------------------------
PSYRATS_total_Baseline |    .976256   .1286083     7.59   0.000     .7219083    1.230604
            _ICentre_1 |  -.3491964   1.066414    -0.33   0.744    -2.458235    1.759842
         _IWorryscor_1 |   -.650908   1.004881    -0.65   0.518    -2.638254    1.336438
         _ICenXWor_1_1 |    .960855   1.396905     0.69   0.493    -1.801793    3.723503
             _Itreat_1 |  -2.380408   .8214766    -2.90   0.004    -4.005036   -.7557804
     MAP_AlcoholAmount |  -.0339031   .0678673    -0.50   0.618    -.1681238    .1003176
         _ItreXMAP_A_1 |   .0585786   .1021132     0.57   0.567    -.1433699     .260527
                 _cons |   -1.31187     2.2432    -0.58   0.560     -5.74823    3.124489
---------------------------------------------------------------------------------------

. xi: regress PSYRATS_total_8WEEK PSYRATS_total_Baseline i.Centre*i.Worryscorestrata
i.treat*WASI_IQ
i.Centre          _ICentre_0-1        (naturally coded; _ICentre_0 omitted)
i.Worryscores~a   _IWorryscor_0-1     (naturally coded; _IWorryscor_0 omitted)
i.Cen~e*i.Wor~a   _ICenXWor_#_#       (coded as above)
i.treat           _Itreat_0-1         (naturally coded; _Itreat_0 omitted)
```

```
i.treat*WASI_IQ   _ItreXWASI__#       (coded as above)

      Source |       SS       df       MS              Number of obs =     133
-------------+------------------------------           F(  7,   125) =   11.75
       Model |  1294.66867     7  184.952668           Prob > F      =  0.0000
    Residual |  1968.15839   125  15.7452671           R-squared     =  0.3968
-------------+------------------------------           Adj R-squared =  0.3630
       Total |  3262.82707   132  24.7183869           Root MSE      =   3.968

------------------------------------------------------------------------------------
   PSYRATS_total_8WEEK |      Coef.   Std. Err.      t    P>|t|     [95% Conf. Interval]
-----------------------+------------------------------------------------------------
PSYRATS_total_Baseline |   .9575123   .1277266     7.50   0.000     .7047255    1.210299
             _ICentre_1 |  -.3682803   1.096509    -0.34   0.738    -2.538408    1.801847
         _IWorryscor_1 |  -1.023627   1.003416    -1.02   0.310    -3.009511    .9622568
         _ICenXWor_1_1 |   1.485486   1.427341     1.04   0.300      -1.3394    4.310371
             _Itreat_1 |   4.663857   3.960414     1.18   0.241    -3.174295    12.50201
               WASI_IQ |   .0295124   .0276824     1.07   0.288    -.0252745    .0842993
         _ItreXWASI__1 |  -.0673473   .0385116    -1.75   0.083    -.1435666     .008872
                 _cons |  -4.039882   3.913494    -1.03   0.304    -11.78517    3.705408
------------------------------------------------------------------------------------

. exit, clear
```
